# Maintenance of spatial gene expression by Polycomb-mediated repression after formation of a vertebrate body plan

**DOI:** 10.1242/dev.178590

**Published:** 2019-10-01

**Authors:** Julien Rougeot, Naomi D. Chrispijn, Marco Aben, Dei M. Elurbe, Karolina M. Andralojc, Patrick J. Murphy, Pascal W. T. C. Jansen, Michiel Vermeulen, Bradley R. Cairns, Leonie M. Kamminga

**Affiliations:** 1 Radboud University, Faculty of Science, Department of Molecular Biology, Radboud Institute for Molecular Life Sciences, Nijmegen 6525 GA, The Netherlands; 2 Radboud University Medical Center, Department of Molecular Biology, Nijmegen 6525 GA, The Netherlands; 3 Howard Hughes Medical Institute, Department of Oncological Sciences and Huntsman Cancer Institute, University of Utah School of Medicine, Salt Lake City, UT 84112, USA; 4 Wilmot Cancer Institute, Rochester Center for Biomedical Informatics, University of Rochester Medical Center, Rochester, NY 14642, USA; 5 Radboud University, Faculty of Science, Department of Molecular Biology, Radboud Institute for Molecular Life Sciences, Oncode Institute, Nijmegen 6525 GA, The Netherlands

**Keywords:** Polycomb, Ezh2, Zebrafish, ChIP-seq, Transcriptomics, Proteomics

## Abstract

Polycomb group (PcG) proteins are transcriptional repressors that are important regulators of cell fate during embryonic development. Among them, Ezh2 is responsible for catalyzing the epigenetic repressive mark H3K27me3 and is essential for animal development. The ability of zebrafish embryos lacking both maternal and zygotic *ezh2* to form a normal body plan provides a unique model for comprehensively studying Ezh2 function during early development in vertebrates. By using a multi-omics approach, we found that Ezh2 is required for the deposition of H3K27me3 and is essential for proper recruitment of Polycomb group protein Rnf2. However, despite the complete absence of PcG-associated epigenetic mark and proteins, only minor changes in H3K4me3 deposition and gene and protein expression occur. These changes were mainly due to local dysregulation of transcription factors outside their normal expression boundaries. Altogether, our results in zebrafish show that Polycomb-mediated gene repression is important immediately after the body plan is formed to maintain spatially restricted expression profiles of transcription factors, and we highlight the differences that exist in the timing of PcG protein action between vertebrate species.

## Abstract (precis)

**Summary:** Our unique zebrafish model of a maternal and zygotic mutant for the Polycomb group gene *ezh2* reveals major conserved and divergent mechanisms in epigenetic gene repression during vertebrate development.

## INTRODUCTION

Development of multi-cellular organisms involves highly dynamic and controlled processes during which one single totipotent cell will multiply and differentiate into all the cells composing the adult individual. Specification of cell identity is controlled through the establishment of spatially and temporally restricted transcriptional profiles, which are subsequently maintained by epigenetic mechanisms (Brock and Fisher, 2005). Epigenetic maintenance of gene expression can act through modifications of the chromatin, the complex of DNA wrapped around an octamer of histones H2A, H2B, H3 and H4, and its associated proteins and non-coding RNAs, creating an epigenetic landscape, often referred to as the epigenome (Zhu and Li, 2016). These modifications can be propagated from mother to daughter cells and thereby maintain gene expression profiles by controlling the accessibility of the DNA to the transcriptional machinery (Li and Reinberg, 2011).

Polycomb Group (PcG) proteins are important regulators of the epigenome during development. First identified in *Drosophila melanogaster*, PcG proteins were found to maintain the pre-established pattern of Hox gene expression (Kennison, 1995). Subsequent studies showed that PcG proteins are important for proper patterning during early embryonic development, tissue-specific development and maintenance of the balance between pluripotency and differentiation of stem cells in multiple species (Schuettengruber et al., 2017). Two main PcG complexes have been described (Chittock et al., 2017). The Polycomb Repressive Complex 2 (PRC2) is composed of the core subunits: EZH1/2 (Enhancer of Zeste Homologue 1/2), SUZ12 (Suppressor of Zeste 12) and EED (Embryonic Ectoderm Development). EZH2 has a catalytically active SET domain that trimethylates lysine 27 of histone H3 (H3K27me3), an epigenetic mark associated with gene repression and found mainly at the transcriptional start sites of gene coding sequences (Mikkelsen et al., 2007). The catalytic subunits of PRC2 are mutually exclusive and EZH1 is postulated to complement the function of EZH2 in non-proliferative adult organs (Margueron et al., 2008; Shen et al., 2008). H3K27me3 can be recognized by the Polycomb Repressive Complex 1 (PRC1). A diversity of PRC1 compositions has been described and canonical PRC1 is composed of the core subunits RING1/RNF2 (Ring Finger Protein 2 a/b), PCGF1-6 (Polycomb Group RING fingers 1-6), PHC (Polyhomeotic) and CBX (Chromobox homolog) (Gao et al., 2012; Kloet et al., 2016). PRC1 catalyzes the ubiquitylation of lysine 119 of histone H2A (H2AK1119ub), and promotes chromatin compaction and subsequent gene repression. In contrast to this canonical view, recent studies suggest that PRC1 is also active in the absence of PRC2 (He et al., 2013; Loubiere et al., 2016; Tavares et al., 2012). Trithorax Group (TrxG) proteins antagonize PcG protein function through the deposition of a trimethyl group on lysine 4 of histone H3 (H3K4me3) on promoters and enhancers from virtually all transcribed genes (Klymenko and Müller, 2004; Santos-Rosa et al., 2002; Schmitges et al., 2011).

In mice, loss of PRC2 genes *Ezh2*, *Eed* or *Suz12* or of the PRC1 gene *Rnf2* leads to post-implantation embryonic lethality during early gastrulation (Faust et al., 1998; O'Carroll et al., 2001; Pasini et al., 2004; Voncken et al., 2003), making it difficult to study transcriptional regulation by PcG complexes during early development. Apart from the mouse model, very few studies have focused on characterization of PcG function during vertebrate development. Lately, the zebrafish embryo has emerged as a model of choice for studying developmental epigenetics in vertebrates (Chrispijn et al., 2019; Lindeman et al., 2011; Murphy et al., 2018; Potok et al., 2013; Vastenhouw et al., 2010). We and others previously used loss-of-function mutants to show that *ezh2* is essential for zebrafish development (Dupret et al., 2017; San et al., 2018, 2016; Zhong et al., 2018). More particularly, our unique vertebrate model of zebrafish embryos mutant for both maternal and zygotic *ezh2*, referred to as *MZezh2* mutant embryos, develop seemingly normally until 1 dpf, forming a proper body plan. These mutants ultimately die at 2 dpf, exhibiting a 100% penetrant pleiotropic phenotype associated with a loss of tissue maintenance (San et al., 2016). This makes zebrafish *MZezh2* mutant embryos a valued model for studying the function of Ezh2 during early development, from fertilization to tissue specification. Furthermore, these mutant embryos provide a unique example of a vertebrate system in which trimethylation of H3K27 has never occurred, unlike cell culture, conditional or zygotic mutant models.

We conducted a multi-omics approach in these *MZezh2* mutant embryos to study how PcG-mediated gene regulation controls axis formation and tissue specification. We focused our study on 24 h post fertilization (hpf) embryos, when the first phenotypes become visible, and the anterior-posterior patterning of the embryos is properly established. Our results show conservation of basic PcG recruitment and silencing mechanisms and reveal that PRC2 function is essential for proper Rnf2 recruitment. However, very surprisingly, the transcriptional and proteomic profile of *MZezh2* mutant embryos remains largely unchanged compared with wild-type embryos, despite the complete absence of Ezh2 protein and its associated epigenetic mark on the chromatin. The changes affect primarily a subset of PcG target genes. These genes are mainly transcription factors essential for developmental processes that present locally restricted aberrant gene expression. Our results show that zebrafish embryo development is initially independent of PcG repression until the stage of tissue maintenance and stress the differences that exist in the timing of PcG function requirement between vertebrate species.

## RESULTS

### The repressive epigenetic mark H3K27me3 is absent in *MZezh2* embryos

**Fig. 1.**
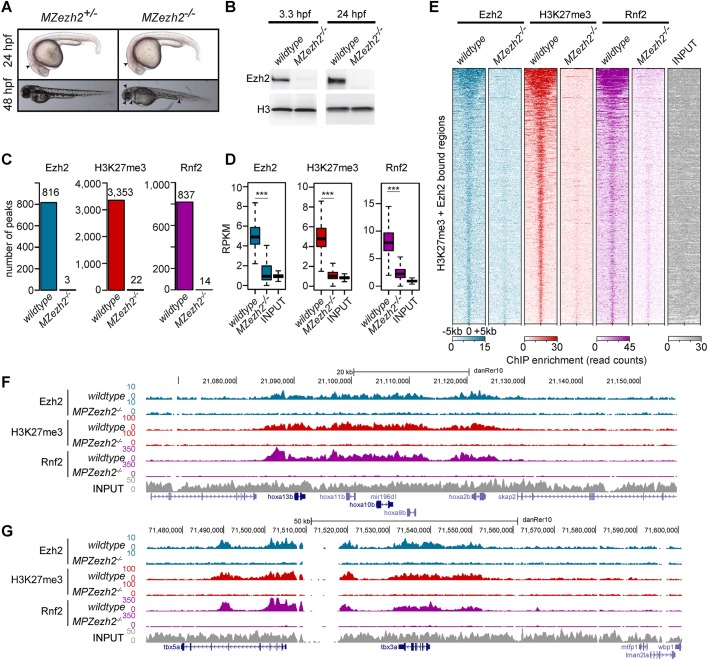
***MZezh2* mutant (*MZezh2^−/−^*) embryos lack Ezh2, H3K27me3 and Rnf2 binding to the chromatin.** (A) *MZezh2^+/−^* (developing as wild-type embryos) and *MZezh2^−/−^* embryos at 24 and 48 hpf. At 24 hpf, *MZezh2^−/−^* embryos lack a clear mid-hindbrain boundary compared with heterozygous embryos (arrowhead). At 48 hpf, *MZezh2^−/−^* embryos showed pleiotropic phenotypes compared with heterozygous embryos, such as small eyes, small brain, heart edema and blood accumulation in the blood island (arrowheads). (B) Western blot analysis of Ezh2 at 3.3 hpf and 24 hpf of wild-type and *MZezh2^−/−^* embryos. Histone H3 was used as a loading control. Results presented are representative of three biological replicates. (C) Number of peaks called after Ezh2, H3K27me3 and Rnf2 ChIP-seq of wild-type and *MZezh2^−/−^* embryos at 24 hpf. Each peak set was obtained by the intersection of two independent biological replicates. (D) Box plots of Ezh2, H3K27me3 and Rnf2 RPKM-normalized coverage after respective ChIP-seq in wild-type and in *MZezh2^−/−^* embryos at 24 hpf. The input control was obtained from wild-type embryos at 24 hpf. Coverages were calculated based on peaks detected in wild-type embryos. ***P<0.001 (*t*-test). The box represents the first quartile, median and third quartile. The whiskers below and above the box represent the minimum and maximum values. (E) Heatmaps for Ezh2, H3K27me3 and Rnf2 subsampled counts after ChIP-seq in 24 hpf wild-type and *MZezh2^−/−^* embryos. Heatmaps are ordered based on coverage intensity in Ezh2 and H3K23me3 ChIP-seq performed in wild types. Windows of 10 kb regions for all H3K27me3 or Ezh2 peaks in 24 hpf wild-type embryos are shown. The input track obtained from 24 hpf wild-type embryos was used as control and was not subsampled. (F,G) UCSC genome browser snapshot depicting the loss of Ezh2, H3K27me3 and Rnf2 after ChIP-seq in 24 hpf *MZezh2^−/−^* embryos compared with wild-type embryos for (F) the *hoxab* gene cluster and (G) the *tbx5a* gene. Colors represent ChIP-seq for different proteins: blue, Ezh2; red, H3K27me3; purple, Rnf2; gray, input control.

To study the function of Ezh2 during development, we used the *ezh2* nonsense mutant allele *ezh2 (hu5670)* containing a premature stop codon within the catalytic SET domain, resulting in the absence of Ezh2 protein (San et al., 2016). Total elimination of both maternal and zygotic contribution of Ezh2 protein and mRNA, by using the germ cell transplantation technique described previously (Ciruna et al., 2002; San et al., 2016), allowed us to study the function of Ezh2 during early development. As previously shown, *MZezh2* mutant embryos display normal body plan formation and a mild phenotype at 24 hpf. They die at 48 hpf, at which point pleiotropic phenotypes are observed, such as smaller eyes, smaller brain, blood coagulation and absence of pectoral fins (Fig. 1A). Western blot analysis at 3.3 hpf and 24 hpf confirmed the absence of both maternal and zygotic Ezh2 in these mutants, respectively (Fig. 1B; Fig. S1). In addition, our previous study also reported that H3K27me3 was not detectable in *MZezh2* mutants by immunofluorescence (San et al., 2016).

To further confirm the absence of Ezh2 in *MZezh2* mutants and its effect on H3K27me3 deposition, we performed ChIP-sequencing (ChIP-seq) for Ezh2 and H3K27me3 at 24 hpf in both wild-type and *MZezh2* mutant embryos. ChIP-seq analyses for Ezh2 and H3K27me3 revealed 816 and 3353 peaks, respectively, in wild-type embryos (Fig. 1C; Table S1). Although the number of peaks differed between the two proteins, their binding profiles greatly overlapped (Fig. 1E). Quantification showed that 85% of Ezh2 peaks also contain H3K27me3 (Fig. S2A). Known PcG target genes, such as the *hoxab* gene cluster, Tbx genes, *isl1* and *gsc* loci presented similar binding profiles for Ezh2 as for H3K27me3 (Fig. 1F,G; Fig. S2B), whereas the ubiquitously expressed genes *eif1ad* and *tbp* showed absence of both Ezh2 and H3K27me3 (Fig. S2B).

In *MZezh2* mutant embryos, the binding of Ezh2 and H3K27me3, as detected by ChIP-seq, was virtually absent, with three and 22 peaks detected for Ezh2 and H3K27me3, respectively (Fig. 1C). Manual inspection of these remaining peaks revealed that they are present in gene deserts and low complexity regions, and are most probably artefacts (Fig. S2B). Ezh2 and H3K27me3 coverage was reduced to background levels in *MZezh2* mutants compared with wild type (Fig. 1D). Finally, the *hoxab* gene cluster, *tbx3a*, *tbx5a*, *gsc* and *isl1* loci, targeted by PcG repression in wild types, also showed a complete absence of Ezh2 and H3K27me3 binding in *MZezh2* mutants (Fig. 1F,G; Fig. S2B).

In order to verify that the absence of detection of Ezh2 and H3K27me3 in *MZezh2* mutant samples was not due to an inefficient ChIP-seq or a normalization artifact specific to mutant samples, the second ChIP-seq replicates for both Ezh2 and H3K27me3 were conducted with spike-in chromatin control. After normalization using the immunoprecipitated spike-in chromatin, the decreases in Ezh2 and H3K27me3 coverage in mutants compared to wild types appear even more pronounced than without spike-in normalization, both at the genome-wide level (Fig. S3A,B) as well as on target genes (Fig. S3C).

Altogether, these results demonstrate that in *MZezh2* mutants Ezh2 and H3K27me3 are absent from the chromatin.

### Loss of PRC2-mediated repression results in impaired PRC1 recruitment during early development

It is postulated that PRC1 is recruited to chromatin by PRC2-deposited H3K27me3 but can also have a function independent of PRC2 (He et al., 2013; Loubiere et al., 2016; Tavares et al., 2012). As both Ezh2 and H3K27me3 are absent from *MZezh2* mutant embryos, we investigated whether PRC1 is still recruited to chromatin in these mutants. In zebrafish, Rnf2 is the only catalytic subunit of PRC1 (Le Faou et al., 2011). ChIP-seq for Rnf2 in wild-type embryos at 24 hpf reveals 837 peaks (Fig. 1C; Table S1) that are present in Ezh2- and H3K27me3-positive regions (Fig. 1E). We found that 70% of the Ezh2 peaks were also positive for Rnf2 in wild-type embryos (Fig. S2A).

In *MZezh2* mutant embryos, only 14 binding sites could be detected for Rnf2 (Fig. 1C) and Rnf2 average binding (measured in RPKM) was reduced to background level, as observed for Ezh2 and H3K27me3 binding (Fig. 1D). This loss of Rnf2 was observed at both gene clusters such as *hoxab* (Fig. 1F) and individual transcription factors such as *tbx3a*, *tbx5a*, *isl1* and *gsc* (Fig. 1G; Fig. S2B). Similar to Ezh2 and H3K27me3, Rnf2-remaining peaks in *MZezh2* mutant embryos were detected in intergenic regions with repeat sequences; these are most probably also artefacts (Fig. S2B). Furthermore, H2AK119ub was barely detectable in core histone extracts from *MZezh2* mutant embryos (Figs S2C and S4), suggesting an impaired functional recruitment of canonical PRC1 to the chromatin in the absence of Ezh2.

### Loss of H3K27me3 in *MZezh2* mutant embryos induces gene-specific gain of H3K4me3

As PcG and TrxG complexes are known to have an antagonistic effect on gene expression (Piunti and Shilatifard, 2016), we investigated whether the loss of H3K27me3 in *MZezh2* mutant embryos changed the deposition of H3K4me3, a mark associated with gene activation.

**Fig. 2.**
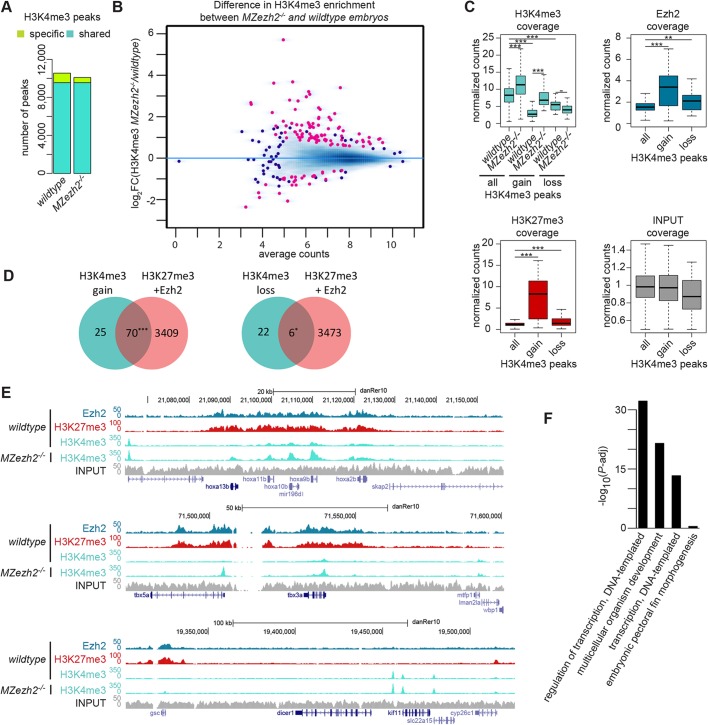
***MZezh2* mutant (*MZezh2^−/−^*) embryos show an increase in H3K4me3 preferentially on H3K27me3 targets.** (A) Number of peaks called after H3K4me3 ChIP-seq in wild-type and *MZezh2* mutant (*MZezh2^−/−^*) embryos at 24 hpf. Turquoise and green represent peaks shared by the two conditions and peaks specific for one condition, respectively. Each peak set was obtained by the intersection of three independent biological replicates. (B) MA plot showing the fold change (log_2_-transformed) in H3K4me3 peak coverages in 24 hpf *MZezh2^−/−^* and wild-type embryos as a function of the normalized average count between the two conditions (log_10_-transformed) as calculated using DiffBind on the union of H3K4me3 peaks detected in both wild-type and *MZezh2* mutant conditions. Red, log_2_FC≥1 or ≤−1 and *P-adj*<0.05; blue, *P-adj*≥0.05. When dot concentration is too high, dots are replaced by density for better visualization. (C) Box plots of subsampled counts after ChIP-seq for H3K4me3 in wild-type and *MZezh2^−/−^* embryos and for Ezh2 and H3K27me3 in wild-type embryos at 24 hpf. Box plots display union of all H3K4me3 peaks detected in *MZezh2^−/−^* or wild-type embryos (all) and H3K4me3 peaks enriched (gain) or decreased (loss) in *MZezh2^−/−^* embryos compared with wild type as detected by DiffBind. Coverages are average of normalized counts between the triplicates for H3K4me3 and duplicates for Ezh2 and H3K27me3. The input track obtained from 24 hpf wild-type embryos was used as a control. ***P<0.001, **P<0.01 (one-way ANOVA with post-hoc tests). The box represents the first quartile, median and third quartile. The whiskers below and above the box represent the minimum and maximum values. (D) Venn diagrams presenting the overlap between peaks with increased or decreased H3K4me3 levels (gain or loss), as detected by DiffBind with the presence of Ezh2 or H3K27me3 peaks within a ±1 kb window. ***P<0.001, *P<0.05 (χ^2^ test). (E) UCSC browser snapshots of three genomic loci in wild-type and *MZezh2^−/−^* embryos at 24 hpf. In C and E, blue, red, turquoise and gray represent ChIP-seq for Ezh2, H3K27me3, H3K4me3 and input control, respectively. (F) Gene ontology analysis of the closest genes restricted to two regions 2 kb upstream or downstream from H3K4me3 peaks enriched in *MZezh2^−/−^*.

To achieve this, we performed ChIP-seq for H3K4me3 in triplicates in both wild-type and *MZezh2* mutant embryos at 24 hpf. We observed a similar distribution of H3K4me3 peaks, with 10,556 peaks detected in wild-type embryos and 10,096 in *MZezh2* mutants (Fig. 2A; Table S1). The majority of the 9550 peaks were shared between wild-type and *MZezh2* mutant embryos (Fig. 2A), suggesting little to no differences in H3K4me3 deposition in absence of Ezh2.

We next assessed the differences in H3K4me3 peak intensity upon loss of Ezh2 by performing differential binding analysis using DiffBind. We identified 95 peaks with an enriched H3K4me3 deposition and 28 peaks with a decreased H3K4me3 intensity in *MZezh2* mutant compared with wild type (Fig. 2B). Analysis of H3K4me3 coverage confirmed the increase of H3K4me3 binding of the sites detected by DiffBind, whereas the decrease in H3K4me3 binding appeared less pronounced (Fig. 2C, upper left panel). Comparisons with Ezh2 and H3K27me3 ChIP-seq showed a clear enrichment in Ezh2 and H3K27me3 binding on the peaks enriched in H3K4me3 in *MZezh2* mutants (Fig. 2C, upper right and lower left panels). The majority of the peaks enriched for H3K4me3 are PcG targets, with 74% (70 out of 95) targeted by Ezh2 or H3K27me3, which is more than expected by chance (*P*-*adj*<0.001). Peaks with decreased H3K4me3 deposition show little enrichment in PcG targets (0.01≤*P*-*adj*<0.05, Fig. 2D) and coverage (Fig. 2C). This result shows that the targets of PcG repression in wild type are more susceptible to presenting an altered H3K4me3 profile upon loss of Ezh2/H3K27me3.

We then searched for the closest genes from the regions with increased H3K4me3 peak coverage detected by DiffBind and identified 118 genes. For example, the transcription factors *hoxa13b*, *tbx5a* and *gsc* showed enrichment for H3K4me3 close to their promoter (Fig. 2E). Gene ontology analysis revealed that these genes were mainly involved in transcriptional regulation and organismal development (Fig. 2F). Among these 118 identified genes, 51 encode for transcription factors, including members of the Hox, Tbx, Sox and Pax gene families, and known targets of PcG complexes. These results show that, at the whole-embryo level, loss of PcG repression has an overall limited effect on the H3K4me3 active epigenetic mark at 24 hpf, and that the genes presenting an increase in H3K4me3 deposition are mainly transcription factors directly targeted by PcG repression.

### Epigenetic changes in *MZezh2* mutant embryos have minor effects on the transcriptome and proteome

The *MZezh2* mutant embryos completely lack the H3K27me3 repressive mark and show a subtle yet selective increased deposition of H3K4me3 activating mark on genes coding for transcription factors. Therefore, we investigated the effect of loss of Ezh2 on the transcriptome and proteome of wild-type and *MZezh2* mutant embryos at 24 hpf.

**Fig. 3.**
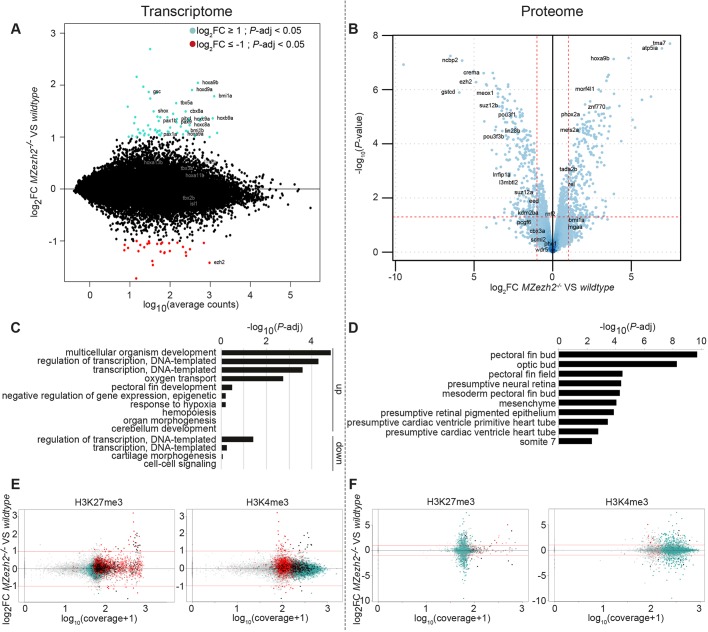
**Loss of maternal zygotic *ezh2* results in overexpression of specific developmental genes.** (A) MA plot showing the fold change (log_2_-transformed) between gene expression in 24 hpf *MZezh2* mutant (*MZezh2^−/−^*) and wild-type embryos as a function of the normalized average count between the two conditions (log_10_-transformed), as calculated with DEseq2. Log_2_FC≥1 and *P-adj*<0.05, turquoise; log_2_FC≤−1 and *P-adj*<0.05, red. For wild-type and *MZezh2^−/−^* embryos, six and seven biological replicates were used, respectively. (B) Volcano plot showing the *P*-value (-log_10_-transformed) as a function of the fold-change (log_2_-transformed) between protein expression level in *MZezh2^−/−^* compared with wild-type embryos at 24 hpf. Data were obtained from biological triplicates for each condition. (C) Gene ontology of biological processes associated with genes upregulated (up) or downregulated (down) in *MZezh2^−/−^* embryos compared with wild-type embryos at 24 hpf. (D) Analysis of anatomical terms associated with proteins upregulated and downregulated in *MZezh2^−/−^* embryos compared with wild-type embryos at 24 hpf. (E,F) Dot plots showing the fold change (log_2_-transformed) between gene expression in 24 hpf *MZezh2^−/−^* and wild-type embryos detected by RNA-seq (E) or proteome analysis (F) as a function of the H3K27me3 (left panel) or H3K4me3 (right panel) coverage [log_10_(coverage+1) transformed]. Red, turquoise, black and gray dots represent genes associated with MACS2-detected peaks for H3K27me3, H3K4me3, both marks or none, respectively.

Transcriptome analysis by RNA-seq in the two conditions revealed only 60 genes significantly upregulated (log_2_FC≥1 and *P-adj*<0.05) and 28 genes downregulated (log_2_FC≤−1 and *P-adj*<0.05) in *MZezh2* mutant compared with wild-type embryos (Fig. 3A; Table S1). We also performed a proteome analysis on whole-embryo extracts in both *MZezh2* mutant and wild-type conditions. This analysis identified 111 upregulated (log_2_FC≥1.5 and *P-adj*<0.05) and 110 downregulated (log_2_FC≤−1.5 and *P-adj*<0.05) proteins in *MZezh2* mutants compared with wild-type controls (Fig. 3B; Table S1).

GO analysis showed that the dysregulated genes in the transcriptomic data are associated with control of organism development and regulation of transcription (Fig. 3C). The proteins dysregulated in the proteome analysis revealed anatomy terms associated with organs that present clear phenotypes or are absent in the *MZezh2* mutant embryos, such as optic bud, heart tube and fins (Fig. 3D). Therefore, it seems like the proteomic analysis reflects the observed phenotypes caused by the disturbed gene expression detected by the transcriptome analysis.

When comparing our RNA-seq results with our ChIP-seq data, we found that upregulated genes are preferentially associated with H3K27me3 (Fig. 3E, left panel), Ezh2 and Rnf2 (Fig. S5A,B) target genes. Quantification showed that 60% (36 out of 60) of the upregulated genes are targets of H3K27me3, which is more than expected by chance (*P*-*adj*<0.001) (Fig. S5C). Interestingly, genes with the higher overexpression are among genes with the higher H3K27me3 coverage (Fig. 3E, left panel). In contrast, upregulated genes show no association with H3K4me3 in wild-type conditions, except for genes decorated by both H3K27me3 and H3K4me3 (Fig. 3E, right panel), but are associated with gain of H3K4me3 in *MZezh2* mutant condition (Fig. S5C). The downregulated genes also show significant association with H3K27me3, but did not show any correlation with gain or loss of H3K4me3 deposition (Fig. 3E; Fig. S5C). This could be explained, for example, by a secondary effect, such as overexpression of a repressor of these genes, or because H3K27me3 could be targeting these genes only in a subset of cells. We cannot distinguish between these potential causes, as experiments were carried out on whole embryos. In contrast, the proteomics data did not present any correlation with either the ChIP-seq or the RNA-seq results (Fig. 3F; Fig. S5D). It appears that proteomic analyses could not detect proteins encoded by H3K27me3 target genes, as demonstrated by the general low H3K27me3 coverage and absence of H3K27me3 targets among the proteins detected by the experiment (Fig. 3F).

Finally, proteome data indicate that, in addition to Ezh2, Suz12b is downregulated in *MZezh2* mutant embryos, whereas other PRC2 core subunits were either not detected or not significantly downregulated (Fig. 3B; Fig. S6). Subunits of the canonical PRC1 complex were mostly not detected or not significantly overexpressed (Fig. S6).

### Ezh2 controls maternal mRNA load in embryos

It is surprising that only a small number of genes are dysregulated upon loss of Ezh2 at the whole-embryo level. One could argue that gene expression levels are more dramatically changed when looking at specific cell populations. We therefore explored gene dysregulation at 0 hpf, before zygotic genome activation, and at 3.3 hpf, when the zygotic genome is activated and cell identity is more homogeneous than at 24 hpf.

We found 1859 upregulated genes and 69 downregulated genes in *MZezh2* mutant embryos when compared with wild-type controls at 3.3 hpf (Fig. S7A). This distribution of dysregulated genes was similar to the results obtained in one-cell stage embryos, when only maternal mRNAs are present, with 1936 genes upregulated and 78 genes downregulated in *MZezh2* mutant embryos compared with wild-type controls (Fig. S7B).

Comparisons between time points show that genes overexpressed in *MZezh2* mutants at 0 and 3.3 hpf greatly overlap, whereas genes overexpressed at 24 hpf are more different (Fig. S7C). However, important transcription factors, such as *gsc*, various Hox genes and *tbx5a*, are dysregulated both at 24 hpf and 0 or 3.3 hpf. GO analysis on genes overexpressed upon loss of Ezh2 at 0 and 3.3 hpf identified specific terms clearly associated with late developmental processes and organogenesis but not with pre-gastrulation events. For example, axon guidance, neural crest cell development and cardiac muscle cell differentiation were among the top terms identified (Fig. S7D). These observations suggest that Ezh2 is important for controlling the load of maternal mRNAs and only later during development to maintain zygotic gene expression.

### Loss of *ezh2* results in expression of Hox genes outside their normal expression domains

We next carried out a spatial expression analysis on selected target genes to distinguish between the possibilities that absence of PcG-mediated repression leads to global but moderate gene dysregulation or to more severe gene dysregulation limited to specific cell types or tissues. We focused on embryos of 24 hpf, when mutants show the first phenotypes without lethality or apoptosis (San et al., 2016).

**Fig. 4.**
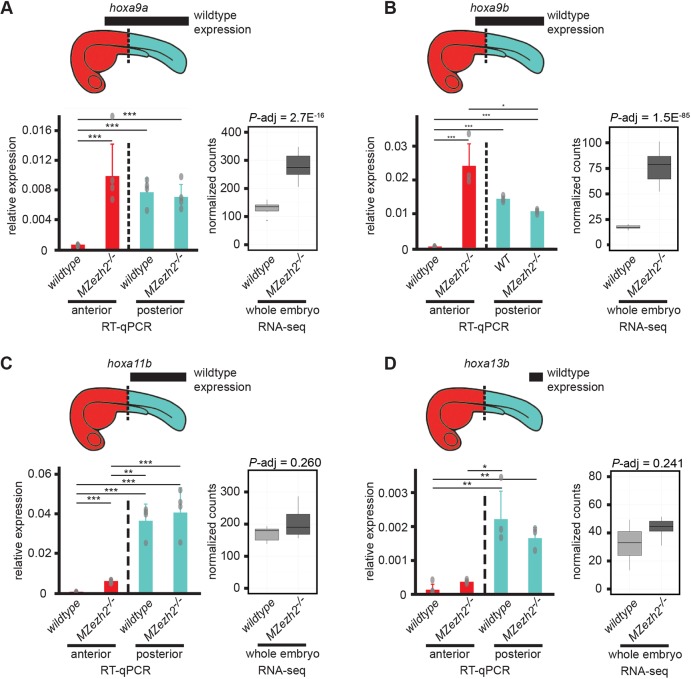
**Loss of maternal and zygotic *ezh2* results in ectopic expression of Hox genes.** (A-D) Expression analysis of (A) *hoxa9a*, (B) *hoxa9b*, (C) *hoxa11b* and (D) *hoxa13b* at 24 hpf. Bar plots on the left side of each panel represent relative expression of indicated Hox genes in the anterior half (red) and posterior half (turquoise) of wild-type and *MZezh2* mutant (*MZezh2^−/−^*) embryos. Boxplots represent normalized counts from RNA-seq experiments in *MZezh2^−/−^* and wild-type whole embryo lysates at 24 hpf. Above is a schematic representation of 1 dpf embryos. Black boxes represent the expression domains of the Hox genes in wild-type embryos based on published data (Thisse, 2004). Dashed lines represent the demarcation between anterior (red) and posterior (turquoise) parts of the embryo used for RT-qPCR analysis. Each experiment was performed at least in triplicate for 20 pooled anterior or posterior larval halves. For RT-qPCR, relative expression was calculated based on expression of the housekeeping gene *actb1*. Data are mean±s.e.m. and overlaid dot plots represent individual RT-qPCR samples. Relative expression was compared between anterior or posterior parts in *MZezh2^−/−^* and wild-type embryos (one-way ANOVA with post-hoc tests, ***P<0.001, **P<0.01, *P<0.05). For RNA-seq, adjusted *P*-values were extracted from differential expression analysis with DEseq2. The box represents the first quartile, median and third quartile. The whiskers below and above the box represent the minimum and maximum values.

To start with, we concentrated on expression of different genes from the Hox gene family. These genes are known targets of Polycomb-mediated repression (Mallo and Alonso, 2013). Every Hox gene has an expression pattern that is restricted along the anterior-posterior axis (Prince et al., 1998). To obtain spatially resolved data along the anterior-posterior axis, we performed RT-qPCR on the anterior half and the posterior half of 24 hpf wild-type and *MZezh2* mutant embryos. We then compared the normalized relative expression levels between the different halves of the *MZezh2* mutant and wild-type embryos. The tested Hox genes were selected based on their domain of expression along the anterior-posterior axis (Fig. 4A-D). The *hoxa9a* gene is expressed mostly in the posterior half of the embryo in wild-type embryos. In *MZezh2* mutant embryos, *hoxa9a* expression increased only in the anterior part, to reach expression levels similar to the wild-type posterior expression (Fig. 4A). No significant differences were detected in the level of expression when comparing the posterior compartment of *MZezh2* mutant and wild-type embryos (Fig. 4A). Similar results were obtained for *hoxa9b*, where overexpression was detected in the anterior compartment of *MZezh2* mutant embryos compared with the anterior compartment of wild-type embryos (Fig. 4B). The *hoxa11b* and *hoxa13b* genes showed higher expression in the posterior half of the wild-type embryos compared with the anterior half (Fig. 4C,D). In the *MZezh2* mutant embryos, both Hox genes were upregulated in the anterior half of the *MZezh2* mutant embryos compared with wild types but their expression level remained lower than in the posterior half of the wild-type embryos (Fig. 4C,D).

Our results are in agreement with previously published data where *hoxa9b*, *hoxd9a*, *hoxc8a* and *hoxc6a* were shown to be ectopically expressed anteriorly in *MZezh2* mutant embryos (San et al., 2016). These comparative analyses of anterior and posterior parts of the embryo suggest that, upon loss of Ezh2, Hox genes show ectopic anterior expression while keeping wild-type expression levels within their normal expression domains.

### Different transcription factors show various profiles of dysregulation in the absence of Ezh2

**Fig. 5.**
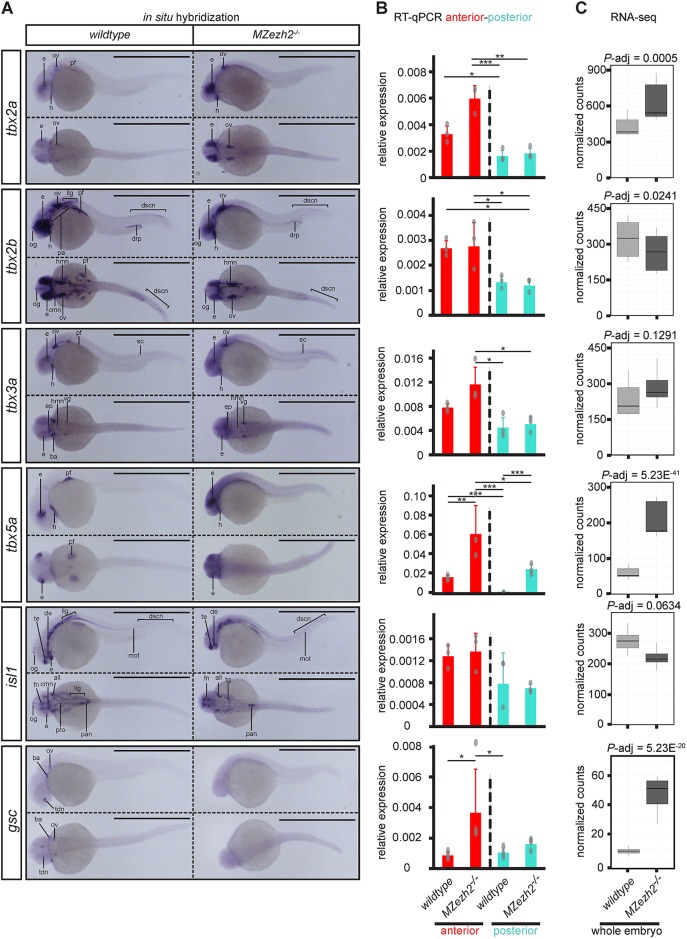
**Transcription factor expression is spatially dysregulated in *MZezh2* mutant (*MZezh2^−/−^*) embryos.** (A-C) Spatial expression analysis by (A) *in situ* hybridization, (B) RT-qPCR on anterior half and posterior half, and (C) RNA-seq results of transcription factors *tbx2a*, *tbx2b*, *tbx3a*, *tbx5a*, *isl1* and *gsc* in 24 hpf embryos. Scale bars: 1 mm. Experiments were performed in biological duplicates of a least 15 pooled embryos for *in situ* hybridization and in triplicates or quadruplicates of 20 pooled larval halves for RT-qPCR. Relative expression was calculated based on expression of the housekeeping gene *actb1*. Data are mean±s.e.m. in B with dots representing individual RT-qPCR samples. Relative expression was compared between anterior (red) or posterior (turquoise) parts in *MZezh2^−/−^* and wild-type embryos (one-way ANOVA with post-hoc tests, ***P<0.001, **P<0.01, *P<0.05). (C) Box plots represent normalized counts from RNA-seq experiments in whole *MZezh2^−/−^* and wild type after differential expression analysis with DEseq2. all, anterior lateral lane ganglion; ba, branchial arch; cmn, cranial motor neurons; de, diencephalon; drp, distal region of the pronephros; dscn, dorsal spinal cord neurons; e, eye; ep, epiphysis; fn, forebrain nuclei; h, heart; hmn, hindbrain motor neurons; llg, lateral lane ganglion; mot, primary motor neurons; og, olfactory ganglion; ov, otic vesicle; pa, pharyngeal arches; pan, pancreas; pf, pectoral fin; pro, pronephros; sc, spinal cord; tdn, telencephalon and diencephalon nuclei; te, telencephalon; vg, ventral ganglion. The box represents the first quartile, median and third quartile. The whiskers below and above the box represent the minimum and maximum values.

To further pursue our investigation on the changes in gene expression patterns in absence of Ezh2, we performed *in situ* hybridization on members from the Tbx gene family of transcription factors. The *tbx2a*, *tbx2b*, *tbx3a* and *tbx5a* genes have partially overlapping expression patterns in wild-type embryos, but also display gene specific expression domains (Fig. 5A). At 24 hpf, these Tbx gene family members are expressed in the dorsal region of the retina, in the heart and the pectoral fins (Ribeiro et al., 2007; Tamura et al., 1999). In addition, *tbx2a*, *tbx2b* and *tbx3a* are expressed in the otic vesicle. The genes *tbx2b* and *tbx3a* are expressed in different ganglions and neurons in anterior and posterior regions of wild-type embryos (Ribeiro et al., 2007). Finally, expression of *tbx2b* can also be detected in part of pharyngeal arches 3-7 and the distal region of the pronephros, and *tbx3a* expression can be detected in the branchial arches (Thisse, 2004). This spatial prevalence of Tbx gene expression in the anterior half of the embryo was also detected by RT-qPCR at 24 hpf, where *tbx2a*, *tbx2b* and *tbx5a* expression was significantly higher in the anterior than in the posterior part of wild-type embryos (Fig. 5B).

*In situ* hybridization for these Tbx genes on *MZezh2* mutant embryos at 24 hpf suggests ectopic expression of these transcription factors around their normal expression pattern in the eye, the otic vesicle and the heart, except for *tbx2b* (Fig. 5A). This scattering in gene expression was reflected in a trend towards a higher expression in the anterior half of *MZezh2* mutant embryos, as detected by RT-qPCR, in which *tbx2a* and *tbx5a* expression showed significant upregulation upon the loss of Ezh2 (Fig. 5B). In addition, *in situ* hybridization for *tbx5a*, and to a lesser extent *tbx3a*, showed ubiquitous expression throughout the entire body of *MZezh2* mutants that was not visible in wild types (Fig. 5A). RT-qPCR results confirmed increased expression of *tbx5a* in both the anterior and posterior half of the *MZezh2* mutant embryos (Fig. 5B).

Besides the observed ectopic expression, all tested Tbx genes showed absence of expression in specific structures upon Ezh2 loss. For example, in *MZezh2* mutant embryos, there are no fin buds formed (San et al., 2016), and there is no expression of all four Tbx genes in the region where the fin buds would normally be present (Fig. 5A). In *MZezh2* mutant embryos, the gene *tbx2b* showed no expression in the pharyngeal arches 3-7 and the lateral line ganglions, and *tbx3a* was not observed in the branchial arches (Fig. 5A). This absence of expression was not detected by RT-qPCR (Fig. 5B) but a trend towards downregulation for *tbx2b* was observed in RNA-seq results on whole *MZezh2* mutant embryo lysates (Fig. 5C).

In addition, we tested transcription factors from other gene families that are targeted by H3K27me3 in wild-type embryos. The transcription factor *isl1*, which is expressed in all primary neurons (Dyer et al., 2014), showed a similar absence of expression in the fin bud and the cranial motor neurons in the midbrain (trigeminal, facial and vagal motor neurons), as observed for *tbx2a*. Its expression was also absent in the ventral region of the eye, the facial ganglia and in the pronephros from *MZezh2* mutant embryos, where it is normally expressed in wild-type embryos (Heisenberg et al., 1999; Zhang et al., 2017) (Fig. 5A). This loss of expression in *MZezh2* mutant embryos was not detected by RT-qPCR but a clear tendency towards downregulation was detected by RNA-seq (Fig. 5B,C). Even more surprising was the expression pattern of *gsc* in the *MZezh2* mutant embryos. Wild-type embryos show highly specific *gsc* expression in the telencephalon and diencephalon nuclei, the branchial arches and the otic vesicle (Thisse, 2004). This expression was lost in *MZezh2* mutant embryos and instead diffuse expression was observed (Fig. 5A). This observation was confirmed by RT-qPCR and RNA-seq, where upregulation of *gsc* was clearly detected in *MZezh2* mutant embryos (Fig. 5B,C).

Taken together, these spatial expression analyses show that the tested transcription factors are expressed outside their normal wild-type expression boundaries in *MZezh2* mutant embryos at 24 hpf. Furthermore, expression of a subset of these genes is lost in specific tissues in the *MZezh2* mutant embryos.

## DISCUSSION

Here, we showed for the first time the genome-wide binding patterns of Ezh2 and Rnf2, the catalytic subunits of PRC2 and PRC1, respectively, in 24 hpf zebrafish embryos. The overall overlap between the binding patterns of Ezh2, Rnf2 and the PcG-related epigenetic mark H3K27me3 suggests that the PcG-mediated gene repression mechanisms (Chittock et al., 2017) are evolutionary conserved in zebrafish development. The complete loss of H3K27me3 in *MZezh2* mutant embryos reveals that Ezh2 is the only methyltransferase involved in trimethylation of H3K27 during early zebrafish development. This result was expected, as Ezh1, the only other H3K27me3 methyltransferase, was shown by a number of studies not to be maternally loaded or expressed in the zebrafish embryo until at least after 1 dpf (Chrispijn et al., 2018; San et al., 2016; Sun et al., 2008; White et al., 2017). In addition, proteomic results showed decreased protein expression of most PRC2 subunits. This could indicate a destabilization of PRC2 in the absence of the catalytic subunit in *MZezh2* mutant embryos. We could therefore confirm that zebrafish embryos can form a normal body plan in the absence of PRC2-mediated gene repression.

The loss of Rnf2 binding in the *MZezh2* mutants suggests that only the canonical pathway, in which PRC2 is required for PRC1 recruitment, is active during this stage of development. This absence of PRC1 recruitment to the chromatin is not caused by an absence of the complex in the *MZezh2* mutants, as most of the PRC1 subunits were detectable and not dysregulated, as shown by proteomic analysis. This is in contrast with studies in cultured mouse embryonic stem cells, where non-canonical PRC1 complexes were shown to be recruited to developmental regulated genes independently of PRC2 (He et al., 2013; Tavares et al., 2012). This difference could be explained by the complete absence of H3K27me3 from fertilization onwards in *MZezh2* mutant embryos; other studies used conditional knockdown. Therefore, our model potentially suggests that the PRC2-independent recruitment of PRC1 during early development can occur if PRC1 recruitment is first primed by a PRC2-dependent mechanism happening earlier during development.

As repressive and activating marks are known to antagonize each other (Schmitges et al., 2011), one could expect an increase in the H3K4me3 level deposited by TrxG proteins in absence of H3K27me3 associated with an increase in gene activation. However, the effects on H3K4me3 deposition, gene expression and protein expression are limited in *MZezh2* mutant embryos at 24 hpf. This observation is in agreement with the near-complete absence of phenotype at this developmental time point. Thus, it appears that transcriptional regulation during zebrafish development is largely PRC2 independent until later stages of development, when maintenance of cellular identity is required. Ezh2, and hence the PRC2 complex, could therefore be responsible for this maintenance, which seems crucial for development and growth. Yet these defects were not associated with apoptosis (San et al., 2016). These results were unexpected, as PRC2 is described to be essential during mammalian development already during gastrulation (Faust et al., 1998; O'Carroll et al., 2001; Pasini et al., 2004). It implies that even if PcG-mediated repression mechanisms are conserved, the developmental stages at which these mechanisms are required differ between species. The external development of the zebrafish and its rapid early development could possibly explain this difference in phenotype.

We also hypothesized that gene dysregulation in the absence of Ezh2 is intense but limited to a subset of cells. To examine this, we performed transcriptome analyses during maternally controlled development (0 hpf) and after zygotic gene activation (3.3 hpf), time points at which the embryo contains one cell or a more homogenous population of cells. First, these transcriptome analyses revealed that Ezh2 is important for controlling the maternal mRNA load transmitted to the embryos. Indeed, in our germ cell transplantation model, the parental females possess mostly wild-type somatic cells but a zygotic *ezh2* (*Zezh2*) mutant germ line. Thus, oogenesis occurs in absence of Ezh2 and leads to the production of oocytes with a modified maternal mRNA load, as reflected by the 0 hpf mutant transcriptome. GO analysis showed that the dysregulated genes belong to developmental pathways normally activated later during development, at the time of organogenesis. It is therefore surprising that the eggs containing a modified maternal mRNA load can mature properly and that the zygote can develop normally until long after the maternally controlled stage of development is over. We hypothesize that these ectopically expressed mRNAs are never translated or that other genes belonging to the same pathways are not expressed, preventing early activation of these late developmental processes.

Second, the comparison of the transcriptome analysis performed at 0 hpf with 3.3 hpf shows that mainly maternal mRNAs are dysregulated. This observation suggests that PRC2-dependent gene repression is not limited to a subset of cells during early development but is rather not required or required only to a very limited extent until 24 hpf.

Although limited, genes that show a gain in H3K4me3 deposition or in expression upon loss of *ezh2* at 24 hpf are mainly transcription factors targeted by H3K27me3 in wild-type embryos. That only a minor fraction of all H3K27me3 target genes gained expression (36 out of 2610=1.2%, Fig. 3E) suggests different mechanisms of regulation of PcG target genes at this time. Our hypothesis is that control of gene expression by signaling pathways and transcription factor networks (McGinnis and Tickle, 2005) is a robust mechanism and can be maintained until 1 dpf in absence of repression by PcG complexes. At 1 dpf, in absence of PcG-mediated repression, the first derepressed genes will be the genes subjected to the most fine-tuned transcriptional control, such as genes controlled by precise morphogen gradients. For example, it has been shown that PRC2 attenuates expression of genes controlled by retinoic acid signaling (Laursen et al., 2013; Zhang et al., 2014). In vertebrates, and most particularly zebrafish, retinoic acid signaling is responsible for induction of formation of, among others, the forelimb field (Cunningham et al., 2013; Grandel and Brand, 2011), dorsoventral patterning of eyes (Lupo et al., 2005; Marsh-Armstrong et al., 1994), hindbrain patterning (Maves and Kimmel, 2005), Hox gene expression (White et al., 2007) and the development of other organs (Samarut et al., 2015). All these processes are affected in *MZezh2* mutant embryos at 24 hpf and onwards, and, therefore, could be explained by a defect in the response to retinoic acid signaling.

Spatial analysis of gene expression revealed different effects on gene expression patterns caused by loss of Ezh2. Anterior-posterior specific RT-qPCR showed that Hox genes become abnormally expressed in the anterior half of the *MZezh2* mutant embryos, whereas expression levels in the posterior half remain unchanged. These results are supported by previous studies showing ectopic expression of Hox genes in PRC1 and PRC2 zebrafish mutants (San et al., 2016; van der Velden et al., 2012), but also in other animal models (Kennison, 1995). Other transcription factors, such as the Tbx gene family members, showed more diverse patterns of dysregulation compared with Hox genes. *In situ* hybridization and RT-qPCR showed that, among the Tbx genes examined, some were overexpressed outside their normal expression domains (*tbx2a*, *tbx3a* and *tbx5a*), whereas others were also ubiquitously upregulated (*tbx3a* and *tbx5a*). The case of eye patterning is a good example of the defect in control of gene expression pattern in *MZezh2* mutant embryos. In wild-type embryos, at 24 hpf, Tbx genes are expressed in the dorsal part of the eye, whereas *isl1* is expressed in the ventral part. Upon loss of Ezh2, our *in situ* hybridization results showed that the expression of the Tbx genes expands to the whole eye, whereas *isl1* disappears from the ventral region. We conclude that Polycomb-mediated repression is therefore responsible for maintenance of expression domains rather than control of expression levels at this time of development in the zebrafish embryo.

Expression analysis by *in situ* hybridization for Hox and Tbx genes as well as for *isl1* also showed loss of expression in specific structures in *MZezh2* mutant embryos. We reasoned that the absence of expression of Hox and Tbx genes in the fin bud is due to the absence of this structure in *MZezh2* mutants (San et al., 2016). The same phenomenon, absence of specific structures, could explain the lack of detection of *tbx2b* and *isl1* in pharyngeal arches, pronephros and lateral line ganglions. The case of *gsc* expression is more striking, as its normal expression pattern is totally abolished and a diffuse expression pattern is detected. The *gsc* gene is known to be expressed in the Spemann organizer during gastrulation and therefore all cells will transiently express *gsc* when undergoing gastrulation (Joubin and Stern, 1999; Stachel et al., 1993). In absence of Ezh2, *gsc* expression could remain active in all cells after leaving the Spemann organizer, leading to a diffuse expression pattern and impaired tissue-specific expression in 24 hpf *MZezh2* mutant embryos.

To conclude, our results show that major characteristics of PcG-mediated repression are conserved in zebrafish, including canonical recruitment or PcG complexes and their function in maintenance of pre-established gene expression patterns. Our use of a mutant depleted of both maternal and zygotic contribution of Ezh2 also reveals that no PRC2-independent recruitment of PRC1 occurs at this stage of development. Finally, we demonstrate that early embryonic development, including germ layer formation and cell fate specification, is independent of PcG-mediated gene repression until axes are formed and organs specified. PcG-mediated gene repression is then required to control precise spatial restricted expression of specific transcription factors. We hypothesize that subtle changes in expression of these important genes subsequently will lead to progressive and accumulating changes in gene network regulation, and result in loss of tissue identity maintenance.

This surprising result highlights the fact that, despite the conservation of PcG-mediated repression mechanisms during evolution, the time frame within which PcG repression is required for proper development may vary greatly between species. Studying the PcG repression in additional species would improve our understanding of the importance of PcG biology during development.

## MATERIALS AND METHODS

### Zebrafish genetics and strains

Zebrafish (*Danio rerio*), were housed according to standard conditions (Westerfield, 2000) and staged according to Kimmel et al. (1995). The *ezh2* nonsense mutant (*hu5670*) (San et al., 2016), *Tg (H2A::GFP)* (Pauls et al., 2001) and *Tg (vas::eGFP)* (Krøvel and Olsen, 2002) zebrafish lines have been described before. Genotyping of the *ezh2* allele was performed as previously described (San et al., 2016) with following adaptations: different primer pairs were used for PCR and nested PCR (Table S2), of which the restriction profile is shown on Fig. S2D. All experiments were carried out in accordance with animal welfare laws, guidelines and policies, and were approved by the Radboud University Animal Experiments Committee.

### Germ cell transplantation

Germ cell transplantation was performed as described previously (San et al., 2016). For all experiments below, *ezh2* germline mutant females were crossed with *ezh2* germline mutant males to obtain 100% *MZezh2* mutant progeny. The germline wild-type sibling males and females obtained during transplantation were used to obtain 100% wild-type progeny with similar genetic background and are referred to as wild type. The embryos used were all from the first generation after germline transplantation.

### Western blotting

At 3.3 hpf, 50 embryos were collected, resuspended in in 500 µl ½ Ringer solution (55 mM NaCl, 1.8 mM KCl, 1.25 mM NaHCO_3_) and forced through a 21 G needle and a cell strainer in order to remove the chorion and disrupt the yolk. At 24 hpf, 20 embryos were collected and resuspended by thorough pipetting in 500 µl ½ Ringer solution in order to disrupt the yolk. The samples of 3.3 and 24 hpf were centrifuged for 5 min at 3500 ***g*** at 4°C and washed two additional times with 500 µl ½ Ringer solution. The embryo pellet was frozen in liquid nitrogen and stored at −80°C. Whole-protein extraction was performed by adding 40 µl of RIPA buffer [100 mM Tris-HCl (pH 8), 300 mM NaCl, 2% NP-40, 1% sodium deoxycholate, 0.2% SDS, 20% glycerol, 1× cOmplete EDTA-free Protease Inhibitor cocktails from Sigma] and sonication for two cycles of 15 s ON and 15 s OFF on medium power at 4°C on a PicoBioruptor (Diagenode). After 10 min incubation at 4°C, embryo lysates were centrifuged for 12 min at 16,000 ***g*** at 4°C and supernatant was transferred in a new tube. Protein (20 µg) was mixed with SDS containing sample loading buffer, denatured at 95°C for 5 min and analyzed by Western blot analysis. Antibodies used for immunoblotting are described in Table S3 HRP-conjugated anti-rabbit secondary antibody was used (Table S3) and protein detection was performed with ECL Select Western Blotting Detection Reagent (GE Healthcare, RPN2235) on an ImageQuant LAS 4000 (GE Healthcare). The anti-H2A western blot was performed on histone extracts, obtained according to van der Velden et al. (2012), and detected on X-ray film. Full uncropped blots used for Fig. 1B and Fig. S2C are available in Figs S1 and S4, respectively.

### ChIP-sequencing

For chromatin preparation, embryos from a germline mutant or germline wild-type incross were collected at 24 hpf and processed per batches of 300 embryos. Embryos were first dechorionated by pronase (0.6 µg/µl) treatment and then extensively washed with E3 medium. Subsequently, embryos were fixed in 1% PFA (EMS, 15710) for 15 min at room temperature and fixation was terminated by adding 0.125 M glycine and washing three times in ice-cold PBS. Yolk from fixed embryos was disrupted by pipetting the fixed embryos 10 times with a 1 ml tip in 600 µl of ½ Ringer solution (55 mM NaCl, 1.8 mM KCl, 1.25 mM NaHCO_3_) and incubated for 5 min at 4°C on a rotating wheel. Embryos were pelleted by centrifuging 30 s at 300 ***g*** and the supernatant was removed. De-yolked embryos were resuspended in 600 µl sonication buffer [20 mM Tris-HCl (pH 7.5), 70 mM KCl, 1 mM EDTA, 10% glycerol, 0.125% NP40, 1× cOmplete EDTA-free Protease Inhibitor cocktails from Sigma] and homogenized with a Dounce homogenizer (six strokes with pestle A, followed by six strokes with pestle B). Homogenates were sonicated for 6 cycles of 30 s ON/30 s OFF on a PicoBioruptor (Diagenode), centrifuged for 10 min at 16,000 ***g*** at 4°C, and the supernatant containing the chromatin was stored at −80°C. Supernatant (20 µl) was subjected to phenol-chloroform extraction and run on an agarose gel to verify that a proper chromatin size of 200-400 bp was obtained.

For ChIP, 100 µl of chromatin preparation (corresponding to 50 embryos) was mixed with 100 µl IP-buffer [50 mM Tris-HCl (pH 7.5), 100 mM NaCl, 2 mM EDTA, 1% NP-40, 1× cOmplete EDTA-free Protease Inhibitor cocktails from Sigma] and antibody (for details on antibodies used, see Table S3) and incubated overnight at 4°C on a rotating wheel. When relevant, *Drosophila* chromatin and anti-H2Av were used according to manufacturer's instructions (Active Motif, 53093 and 61686). For immunoprecipitation, 20 µl of protein G magnetic beads (Invitrogen, 1003D) were washed in IP buffer and then incubated with the chromatin mix for 2 h at 4°C on a rotating wheel. Samples were washed in 500 µl washing buffer 1 (IP-buffer+0.1% sodium deoxycholate), followed by washing in washing buffer 2 (washing buffer 1+400 mM NaCl), washing buffer 3 (washing buffer 1+250 mM LiCl), washing buffer 1 and a final wash in 250 µl of TE buffer. All washes were 5 min at 4°C on a rotating wheel. Chromatin was eluted from the beads by incubation in 100 µl of elution buffer [50 mM NaHCO_3_ (pH 8.8), 1% SDS] for 15 min at 65°C at 900 rpm in a thermomixer. The supernatant was transferred in a clean 1.5 ml tube. Elution was repeated a second time and both supernatants were pooled. The eluate was treated with 0.33 µg/µl RNaseA for 2 h at 37°C. Samples were then decrosslinked by adding 10 µl of 4 M NaCl and 1 µl of 10 mg/ml proteinase K, and incubated overnight at 65°C. DNA was then purified using MinElute Reaction Clean-Up kit (Qiagen, 28204).

DNA (1-5 ng) was used to prepare libraries with the KAPA Hyper Prep Kit (KAPABiosystems, KK8504) and NEXTflex ChIP-Seq Barcodes for Illumina (Bioo Scientific, 514122) followed by paired-end 43 bp sequencing on an Illumina NextSeq500 platform. All ChIP-seq were performed in two biological replicates, except for H3K4me3 in *MZezh2* mutant and wild-type embryos, which were performed in triplicate.

### RNA-sequencing

Ten to 20 manually dechorionated 24 hpf embryos of a germline mutant incross and a germline wild-type incross were homogenized in TRIzol (Ambion, 15596018). For 0 and 3.3 hpf, 20 non-dechorionated embryos were collected and homogenized in Trizol. Subsequently, the Quick RNA microprep kit (Zymo Research, R1051) was used to isolate RNA and treat the samples with DNAseI. Samples were depleted from rRNA using the Ribo-Zero rRNA Removal Kit (Illumina, MRZH11124), followed by fragmentation and cDNA synthesis, and libraries were generated using the KAPA Hyper Prep Kit (KAPABiosystems, KK8504). Sequencing libraries were paired-end sequenced (43 bp read-length) on an Illumina NextSeq500 platform. However, two samples per genotype at 24 hpf were generated with the TruSeq Stranded Total RNA Library Prep Kit with Ribo-Zero (Illumina, RS-122-2201) and single-end sequenced (50 bp read-length) on an Illumina HiSeq 2500. For wild-type and *MZezh2* mutant embryos, six and seven biological replicates were used, respectively.

### Mass spectrometry

At 24 hpf, 50 embryos were collected, dechorionated and resuspended by gently pipetting in 500 µl deyolking buffer (1/2 Ginzburg Fish Ringer without calcium: 55 mM NaCl, 1.8 mM KCl, 1.25 mM NaHCO3, 1× cOmplete EDTA-free Protease Inhibitor cocktail from Sigma) and incubated for 5 min in a Thermomixer at room temperature at 1100 rpm to disrupt the yolk. The samples were then centrifuged for 30 s at 400 ***g*** and the pellet was washed two times in 0.5 ml wash buffer [110 mM NaCl, 3.5 mM KCl, 2.7 mM CaCl_2_, 10 mM Tris/Cl (pH 8.5), 1× cOmplete EDTA-free Protease Inhibitor cocktail from Sigma] for 2 min in a Thermomixer at room temperature and 1100 rpm, followed by 30 s centrifugation at 400 ***g***. Washed pellets were lysed in 100 µl RIPA buffer [50 mM Tris (pH 8.0), 150 mM NaCl, 0.1% SDS, 1% NP-40, 0.5% DOC, 20% glycerol, 1 mM sodium orthovanadate, 1× cOmplete EDTA-free Protease Inhibitor cocktails from Sigma] and sonicated for 2 cycles of 15 s ON and 15 s OFF on full power at 4°C on a Bioruptor (Diagenode). Samples were incubated for 1 h on a rotating wheel at 4°C and centrifuged 10 min at 12,000 ***g*** and 4°C. Supernatant was flash frozen and stored at −80°C. After Bradford analysis, 100 µg protein lysate was used for FASP-SAX as previously described (Wiśniewski et al., 2011). The peptide fractions were separated on an Easy nLC 1000 (Thermo Scientific) connected to a Thermo scientific Orbitrap Fusion Tribrid mass spectrometer. MS and MS/MS spectra were recorded in a top speed modus with a run cycle of 3 s using Higher-energy Collision Dissociation (HCD) fragmentation. The raw mass spectrometry data were analyzed using the MAXQuant software version 1.6.0.1 (Cox and Mann, 2008) with default settings. Data were searched against the *Danio rerio* data base (UniProt June 2017). The experiment was performed with biological triplicates for each condition.

### Bioinformatics analyses

For ChIP-seq analysis, fastq files were aligned to GRCz10 zebrafish genome version using BWA-MEM (version 0.7.10-r789) for paired-end reads (Li and Durbin, 2009). Statistics for all high-throughput sequencing samples generated for this study are presented in Table S4 and Fig. S8. Duplicated and multimapping reads were removed using samtools (Li et al., 2009) version 1.2 and Picard tools (broadinstitute.github.io/picard) version 2.14.1. When spike-in normalization was used, *Drosophila* reads were aligned to dm6 *Drosophila* genome version and a normalization factor was then applied to zebrafish reads according to manufacturer's protocol (Active Motif, 53093 and 61686). MACS2 (Zhang et al., 2008) version 2.1.1 was used to call peaks from each aligned bam files using an Input track from 24 hpf wild-type embryos as control sequence. Peaks separated by less than 1 kb distance were merged, peaks that were called using Input alone were removed from all datasets using bedtools suit version 2.20.1, and the intersection between the replicates for each antibody in each condition was used to define the definitive peak sets. For visualization in heatmaps and genome browser snapshots, fastq files from duplicate ChIP-sequencing were merged, aligned as described above, subsampled to equalized read numbers between wild-type and *MZezh2* mutant conditions for each ChIP, and transformed into bigwig alignment files using bam2bw version 1.25. Peak lists were analyzed using bedtools, and heatmaps were produced using deepTools plotHeatmap (Ramírez et al., 2016) version 2.5.3. Comparison between H3K4me3 peaks in *MZezh2* mutant and wild-type conditions was performed using DiffBind version 2.10.0 on the union between H3K4me3 peaks detected in both conditions.

For RNA-sequencing analysis, read counts per gene were retrieved using GeneCounts quantification method from STAR (Dobin et al., 2013) version 2.4.0 and the GRCz10 zebrafish genome version with Ensembl annotation version 87 as reference. Differential expression analysis was calculated with DESeq2 (Love et al., 2014) version 1.14.1. For proteomics analysis, differential expression of protein between conditions was assessed with DEP (Zhang et al., 2018) version 1.2.0. Gene Ontology analyses on selected genes were performed using DAVID bioinformatics resources (Huang et al., 2009) version 6.8 and anatomical term enrichment was carried out using ZEOGS (Prykhozhij et al., 2013).

### Whole-moun*t in situ* hybridization

Embryos at 24 hpf were dechorionated and fixed overnight at 4°C in 4% PFA in PBST (0.1% Tween), after which they were gradually transferred to 100% methanol. Prior to *in situ* hybridization, embryos were gradually transferred back to PBST and, subsequently, *in situ* hybridization was performed as described previously (Houwing et al., 2007). *In situ* hybridization was performed simultaneously for wild-type and *MZezh2* mutant embryos, with the same probe and chemical mixes, and identical signal development times. The embryos were imaged by light microscopy on a Leica MZFLIII equipped with a DFC450 camera.

### RT-qPCR analyses

Total RNA was isolated using Trizol from 20 flash-frozen dechorionated 24 hpf wild-type and *MZezh2* mutant embryos cut in two with tweezers. Reverse transcription was achieved using Superscript III (Invitrogen, 18080093) and poly-dT primers. Standard qPCR using SYBR Green (iQ SYBR Green Supermix, BioRad, 1708880) was performed using the primers shown in Table S2. Relative expression was calculated based on expression of the housekeeping gene β-actin. Comparable results were obtained using *eif1a* as reference gene (Fig. S9). Calculations were based on at least three independent replicates for both conditions.

## Supplementary Material

Supplementary information
