# Supplementary material for: Maintenance of spatial gene expression by Polycomb-mediated repression after formation of a vertebrate body plan
Source: Development. 2019 Sep 30;146(19):dev178590. doi: 10.1242/dev.178590 (PMC6803366; doi:10.1242/dev.178590)
Supplement: Supplementary information [file develop-146-178590-s1.pdf]

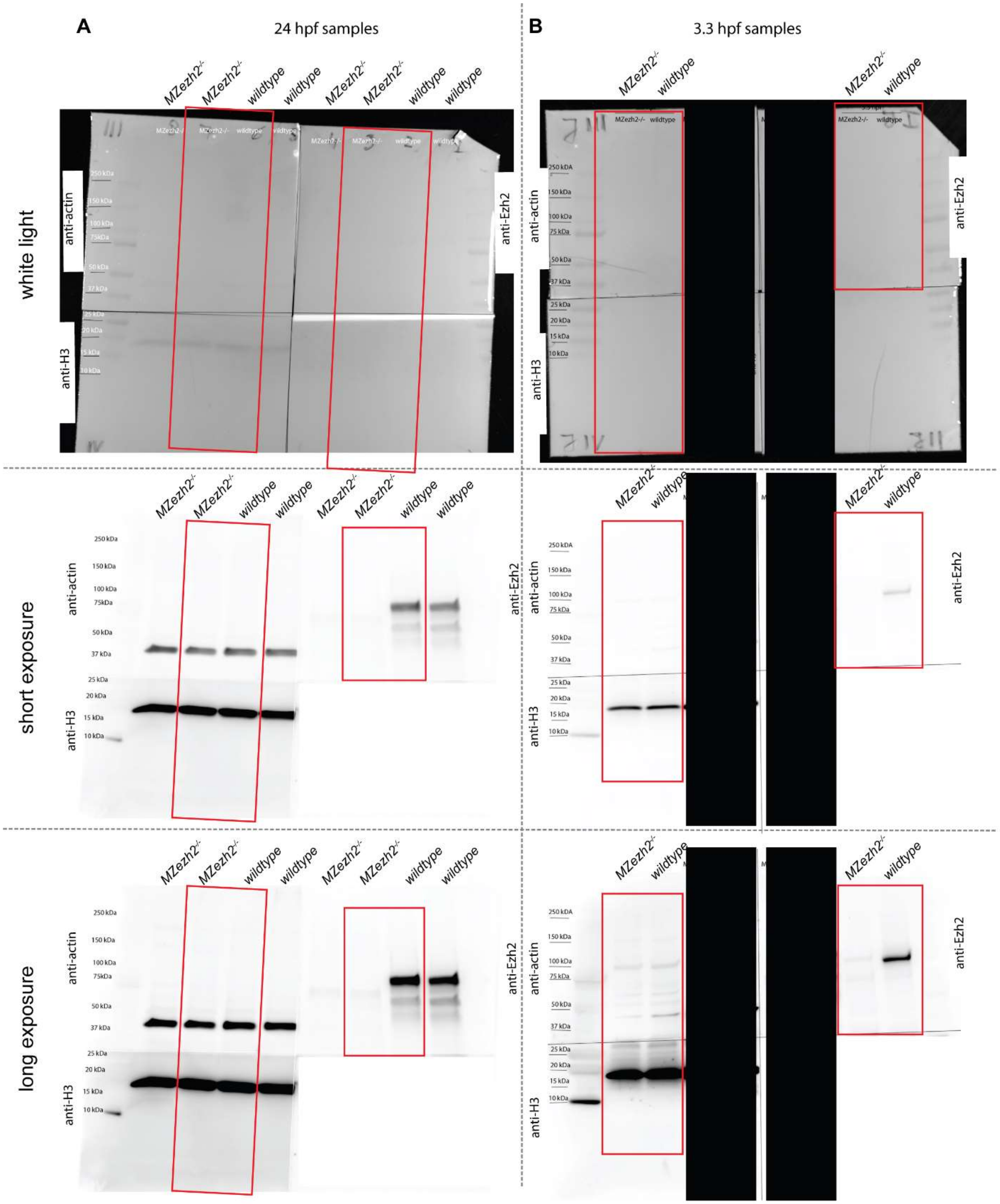

Rougeot_Supplemental_Fig.1

Fig. S1. Uncropped Western blots used for Figure 1B. (**A**) Images of uncropped Western blot taken for detection of Ezh2, H3K27me3, and Histone H3 in wildtype (*MZezh2*$^{+/+}$) and *MZezh2* mutant (*MZezh2*$^{-/-}$) embryos at 24 hpf obtained with white light illumination (top), chemical luminescence for a short exposure time (middle), and chemical luminescence for a longer exposure time

Development • Supplementary information

(bottom). (**B**) Images of uncropped western blot taken for detection of Ezh2, H3K27me3, and Histone H3 in wildtype (*MZezh2*$^{+/+}$) and *MZezh2* mutant (*MZezh2*$^{-/-}$) embryos at 3.3 hpf obtained with white light illumination (top), chemical luminescence for a short exposure time (middle), and chemical luminescence for a longer exposure time (bottom). Red framed lanes correspond to samples shown in Figure 1B. Black boxes cover lanes not used in this study.

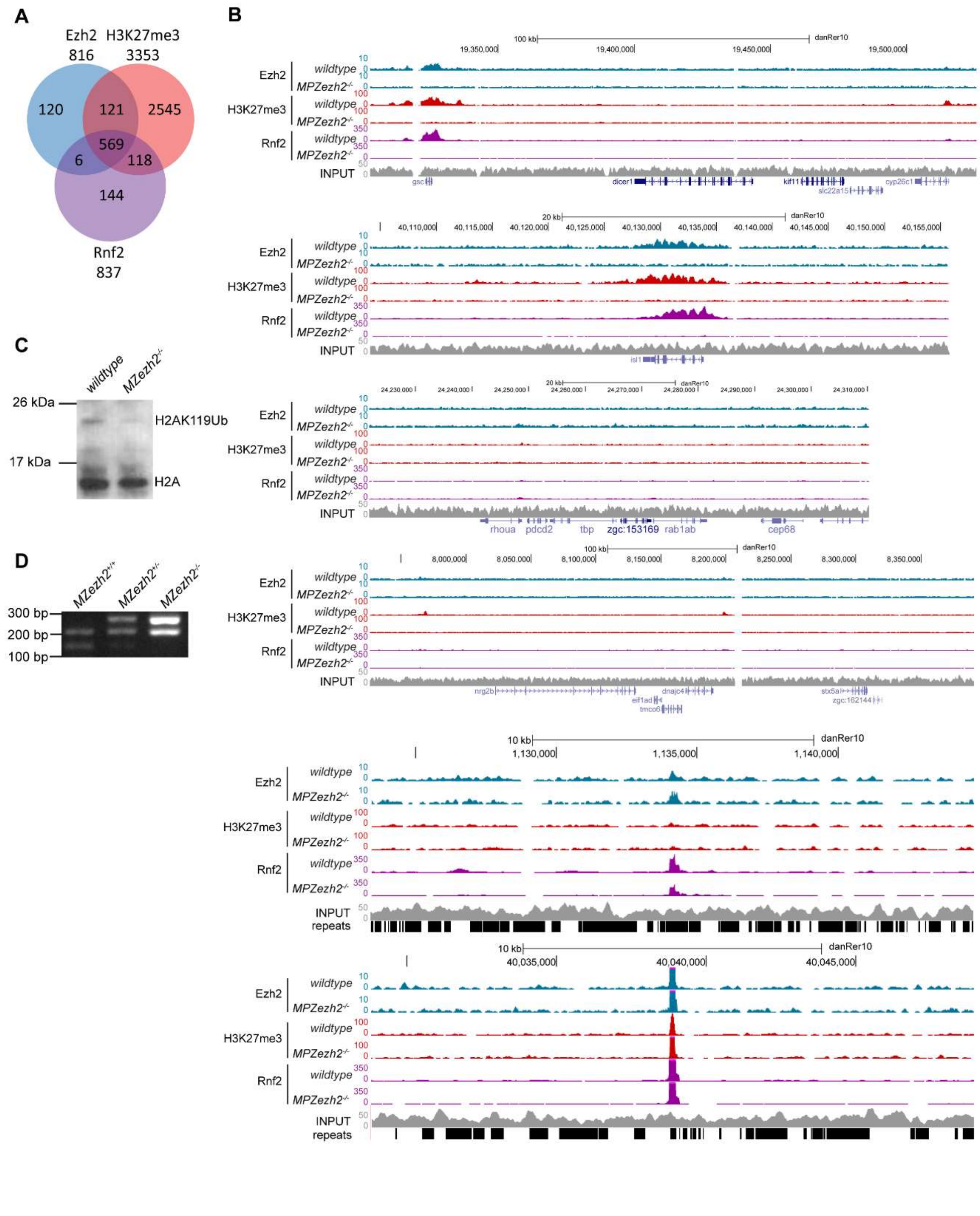

Rougeot_Supplemental_Fig.2

Fig. S2. Analysis of Ezh2, H3K27me3, and Rnf2 binding in wildtype and *MZezh2* mutant (*MZezh2*$^{-/-}$) embryos at 24 hpf. (**A**) Venn diagrams presenting the overlap between Ezh2 (blue), H3K27me3 (red), and Rnf2 (purple) peaks detected in 24 hpf wildtype embryos. (**B**) UCSC browser snapshots of six

Development • Supplementary information

genomic loci depicting Ezh2, H3K27me3, and Rnf2 binding after ChIP-seq in *MZezh2*$^{-/-}$ embryos compared to wildtype embryos at 24 hpf. Colors represent ChIP-seq for different proteins with blue: Ezh2, red: H3K27me3, purple: Rnf2, and grey: Input control. (**C**) Western blot analysis of H2A on histone extracts at 24 hpf in wildtype and *MZezh2*$^{-/-}$ embryos. The presence of H2AK119 monoubiquitylation was visualized as a shift of the H2A band from 13 kDa to ≥20 kDa as showed by van der Velden et al. (2012). Experiment was performed in biological duplicates. (**D**) Example of *ezh2*^{hu5670} genotyping results after nested PCR, RsaI restriction, and gel electrophoresis in *MZezh2* wildtype (*MZezh2*$^{+/+}$), *MZezh2* heterozygous (*MZezh2*$^{+/-}$), and *MZezh2* mutant (*MZezh2*$^{-/-}$) embryos.

Development • Supplementary information

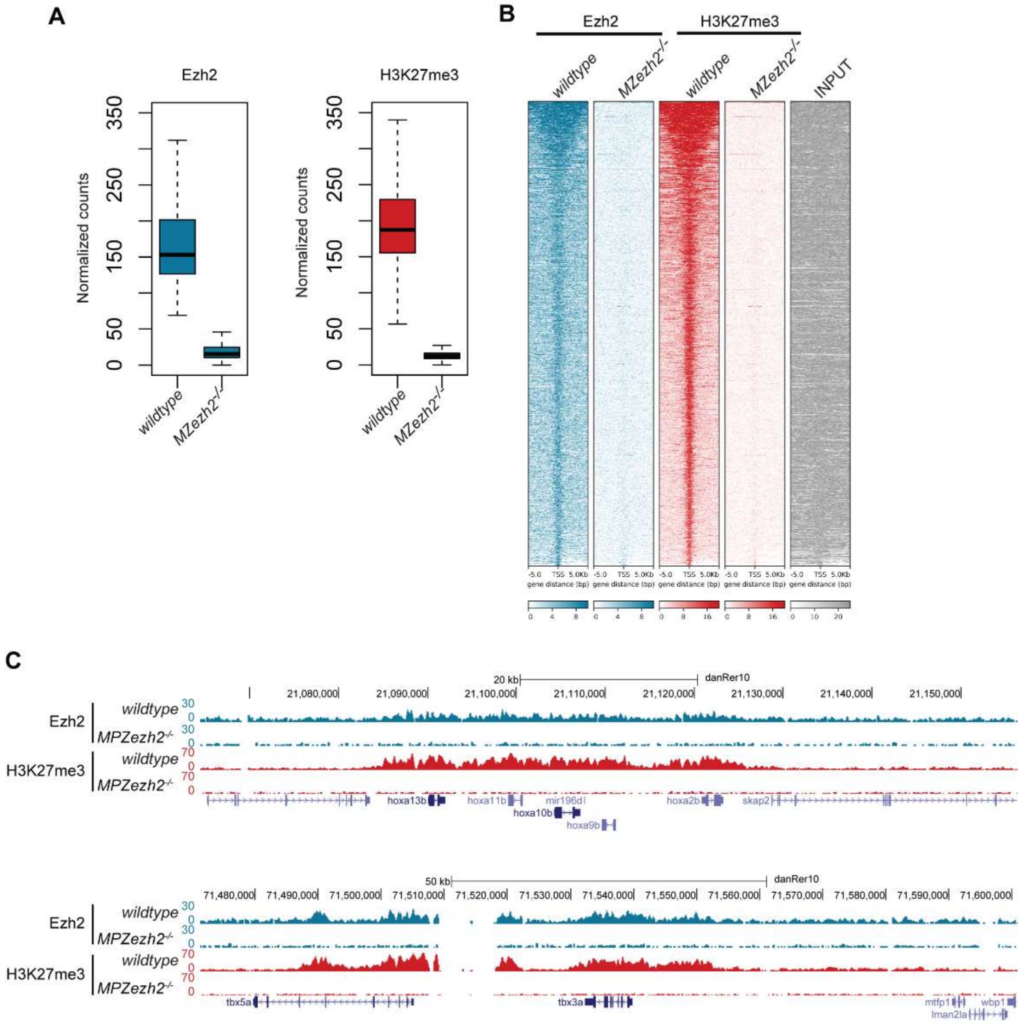

Rougeot_Supplemental_Fig.3

Fig. S3. ChIP-seq of Ezh2 and H3K27me3 using spike-in chromatin for normalization. (**A**) Box plots of Ezh2, H3K27me3, and Rnf2 coverage based on spike-in normalization after ChIP-seq in wildtype and in *MZezh2$^{-/-}$* embryos at 24 hpf. Coverages were calculated based on positions of peaks detected in wildtype embryos. One replicate was performed with spike-in chromatin for each condition. The box represents the first quartile, median and third quartile. The whiskers below and above the box represent the minimum and maximum values. (**B**) Heatmaps for Ezh2, H3K27me3, and Rnf2 counts normalized with spike-in chromatin after ChIP-seq in 24 hpf wildtype and *MZezh2$^{-/-}$* embryos. Windows of 10 kb regions for all H3K27me3 or Ezh2 peaks in 24 hpf wildtype embryos are shown. The input track obtained from 24 hpf wildtype embryos was used as control and was not normalized. (**C**) UCSC genome browser snapshot depicting the loss of Ezh2 and H3K27me3 after ChIP-seq in 24 hpf *MZezh2$^{-/-}$* embryos compared to wildtype embryos. Coverage were normalized with spike-in chromatin.

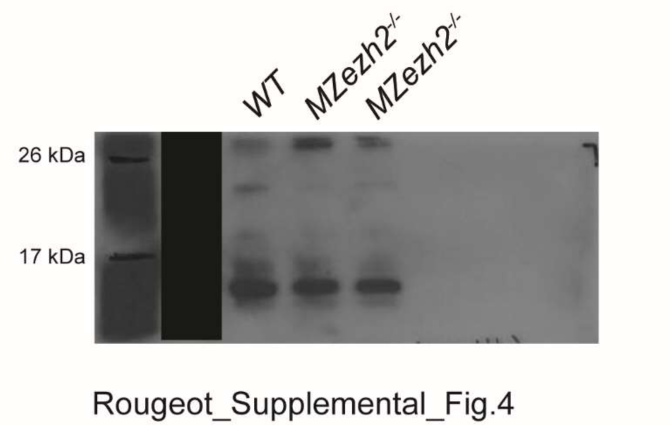

Fig. S4. Uncropped Western blot used for Figure S2C. Image of uncropped Western blot taken for detection of Histone H2A in wildtype (*WT*) and *MZezh2* mutant (*MZezh2*$^{-/-}$) embryos at 24 hpf. The presence of H2AK119 monoubiquitylation was visualized as a shift of the H2A band from 13 kDa to ≥20 kDa as showed by van der Velden et al. (2012). Black box covers data not used in this study.

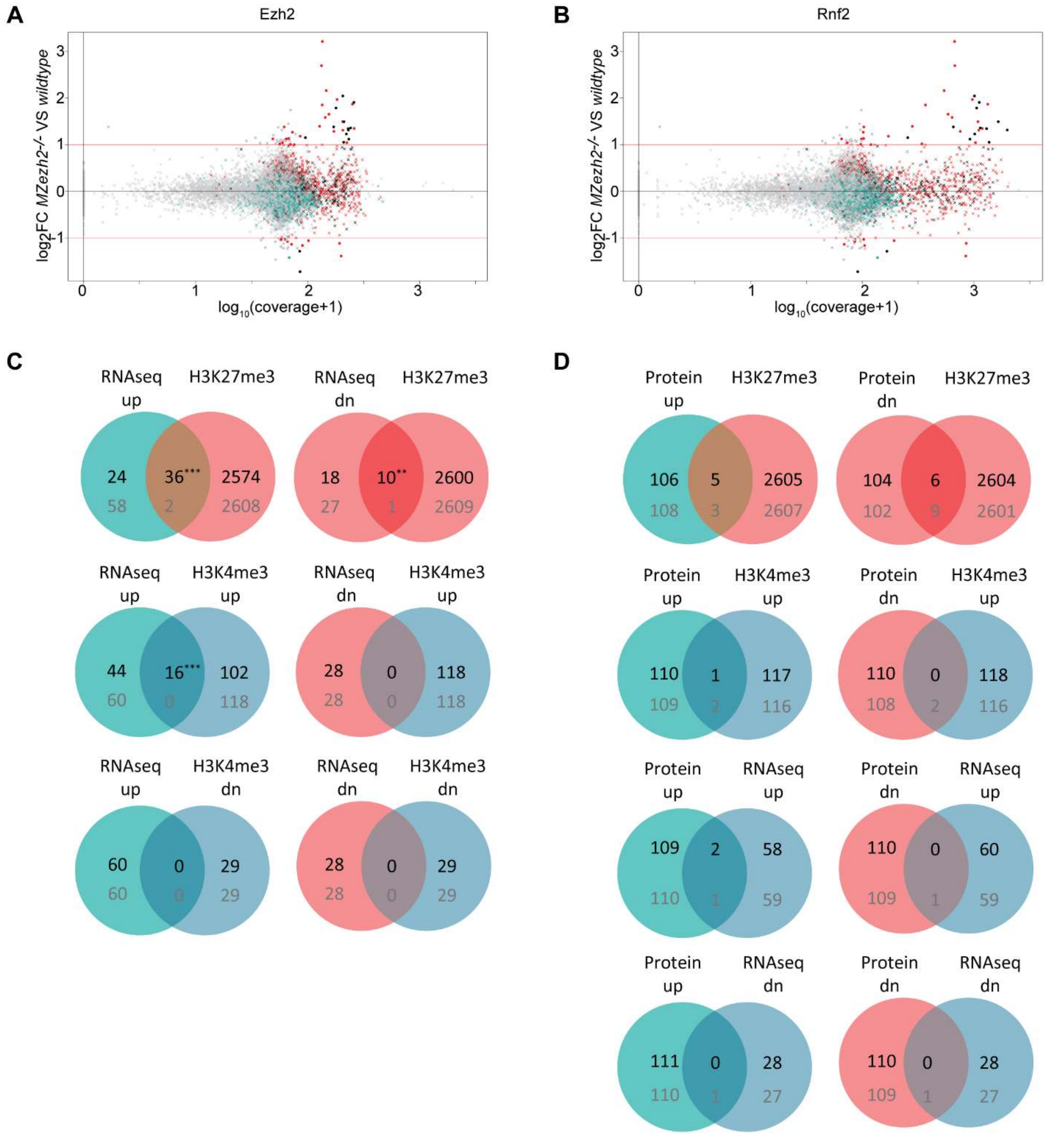

Rougeot_Supplemental_Fig.5

Fig. S5. Integration of ChIP-seq, RNA-seq, and proteomics data. (**A**) Dot plot showing the fold change (log_2-transformed) between gene expression in 24 hpf *MZezh2* mutant (*MZezh2*$^{-/-}$) and wildtype embryos detected by RNA-seq as a function of the Ezh2 coverage (log_{10}(coverage+1) transformed). (**B**) Dot plot showing the fold change (log_2-transformed) between gene expression in 24 hpf *MZezh2*$^{-/-}$ and wildtype embryos detected by RNA-seq as a function of the Rnf2 coverage (log_{10}(coverage+1) transformed). In **A** and **B**, coverage was calculated on the gene region +/- 2 kb and

Development • Supplementary information

averaged between duplicates. (**C**) Venn diagrams presenting the overlap between genes upregulated (up) or downregulated (dn) in *MZezh2*$^{-/-}$ embryos compared to wildtype and presence of H3K27me3 or H3K4me3 peaks. The closest genes from H3K27me3 peaks in wildtype condition or H3K4me3 enriched (H3K4me3 up) and decreased (H3K4me3 dn) peaks according to DiffBind were used for this analysis. Black numbers represent comparison between actual DEseq2 identified genes and closest genes from peaks. Grey numbers represent comparisons between actual DEseq2 identified genes and random selected genes used as control. X^2: *** *P*-value < 0.001, ** *P*-value < 0.01, * *P*-value < 0.05. (**D**) Venn diagrams presenting the overlap between proteins overrepresented (Protein up) or underrepresented (Protein dn) in *MZezh2*$^{-/-}$ embryos compared to ChIP-seq and RNA-seq results. The closest genes from H3K27me3 peaks in wildtype condition or H3K4me3 enriched peaks according to DiffBind (H3K4me3 up) were used for this analysis. Black numbers represent comparison between actual dysregulated proteins and genes. Grey numbers represent comparisons between actual dysregulated proteins and random selected genes used as control. X^2-test did not provide any significant results.

Development • Supplementary information

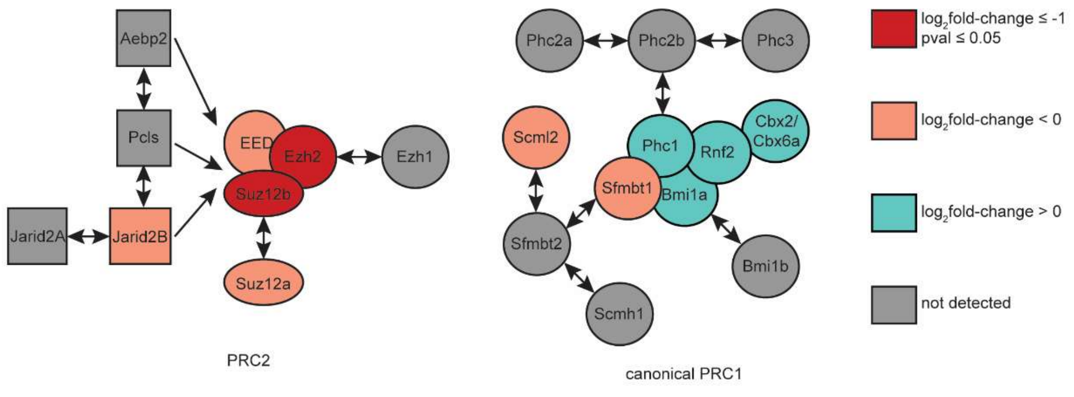

Fig. S6. Proteomic analysis in *MZezh2* mutant (*MZezh2*$^{-/-}$) embryos at 24 hpf reveals downregulation of the core PRC2 components. Schematic representation of changes in protein expression level of PRC2 (left) and canonical PRC1 (right) subunits in *MZezh2*$^{-/-}$ compared to wildtype embryos at 24 hpf. Dark red: $\log_2$fold-change ≤ -1 and *P*-value ≤ 0.05, light red: $\log_2$fold-change < 0, turquoise: $\log_2$fold-change ≥ 0, grey: protein not detected.

Development • Supplementary information

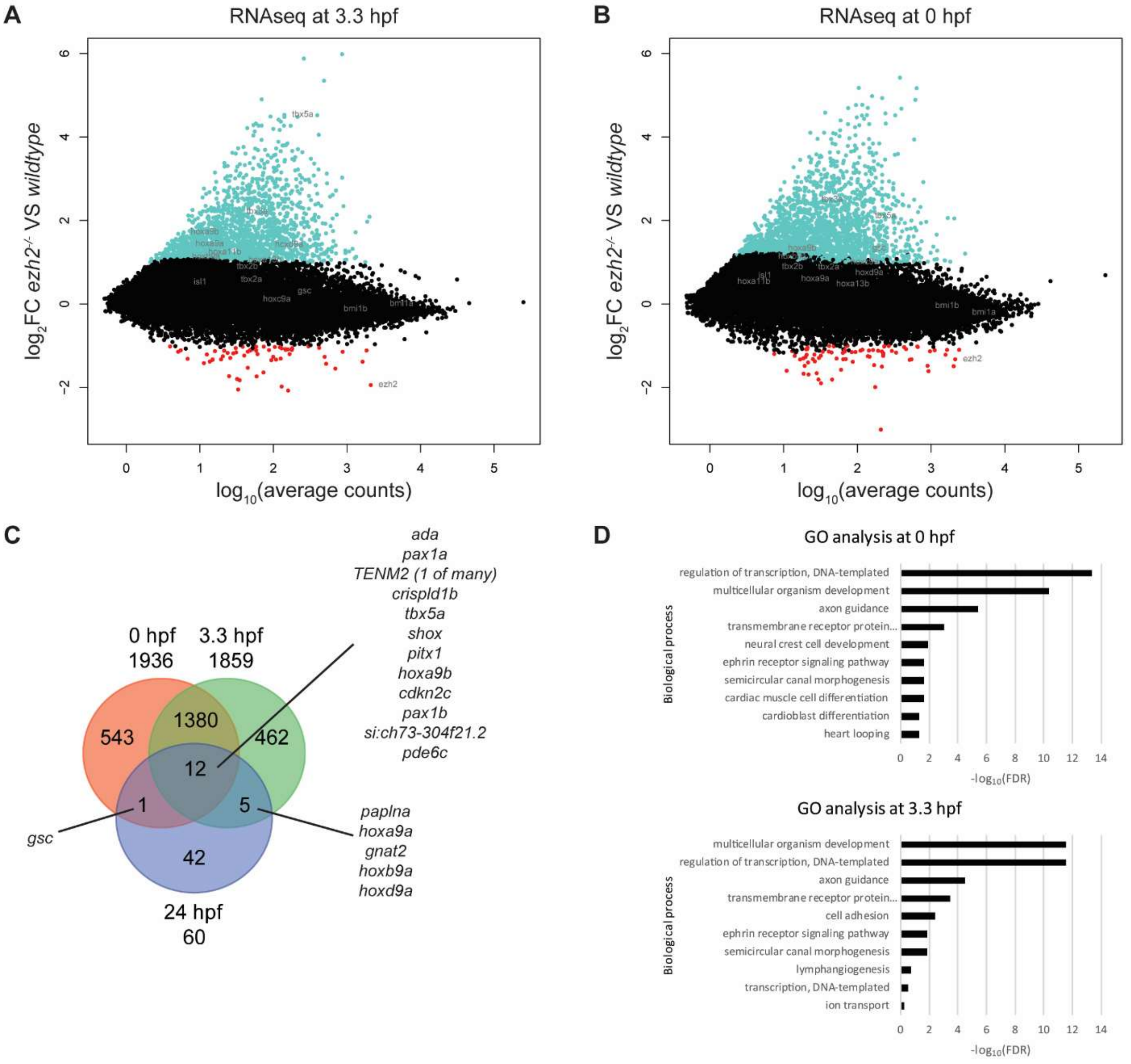

Rougeot_Supplemental_Fig.7

Fig. S7. Transcriptome analysis of *MZezh2* mutant at 3.3 and 0 hpf. (**A**) MA-plot showing the fold change (log_2-transformed) between gene expression in 3.3 hpf *MZezh2* mutant (*MZezh2*$^{-/-}$) and wildtype embryos as a function of the normalized average count between the two conditions (log_{10}-transformed) as calculated with DEseq2. Turquoise: $log_2FC \geq 1$ and *P*-adj < 0.05, red: $log_2FC \leq -1$ and *P*-adj < 0.05. Experiments were performed in at least 5 biological replicates. (**B**) MA-plot showing the fold change (log_2-transformed) between gene expression in 0 hpf *MZezh2*$^{-/-}$ and wildtype embryos as a function of the normalized average count between the two conditions (log_{10}-transformed) as calculated with DEseq2. Turquoise: $log_2FC \geq 1$ and *P*-adj < 0.05, red: $log_2FC \leq -1$ and *P*-adj < 0.05. (**C**) Venn diagram comparing genes overexpressed in *MZezh2*$^{-/-}$ compared with wildtype embryos at 3 different time points. (**D**) Gene Ontology of biological processes associated with genes upregulated in *MZezh2*$^{-/-}$ embryos compared to wildtype embryos at 3.3 and 0 hpf.

Development • Supplementary information

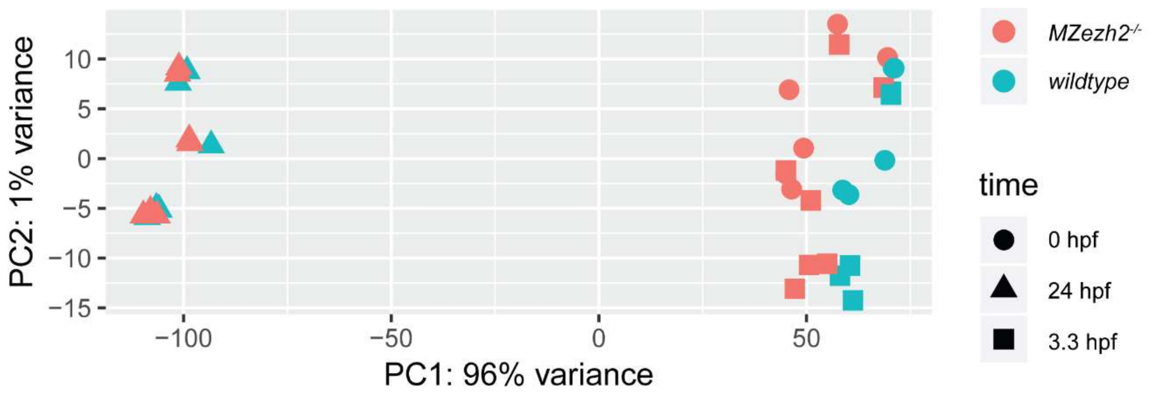

Rougeot_Supplemental_Fig.8

Fig. S8. Principal Component Analysis (PCA) for all RNA-sequencing samples generated for this study. Principal Component (PC) 1 explains most of the variation by clearly separating 24 hpf samples from 0 and 3.3 hpf samples.

Development • Supplementary information

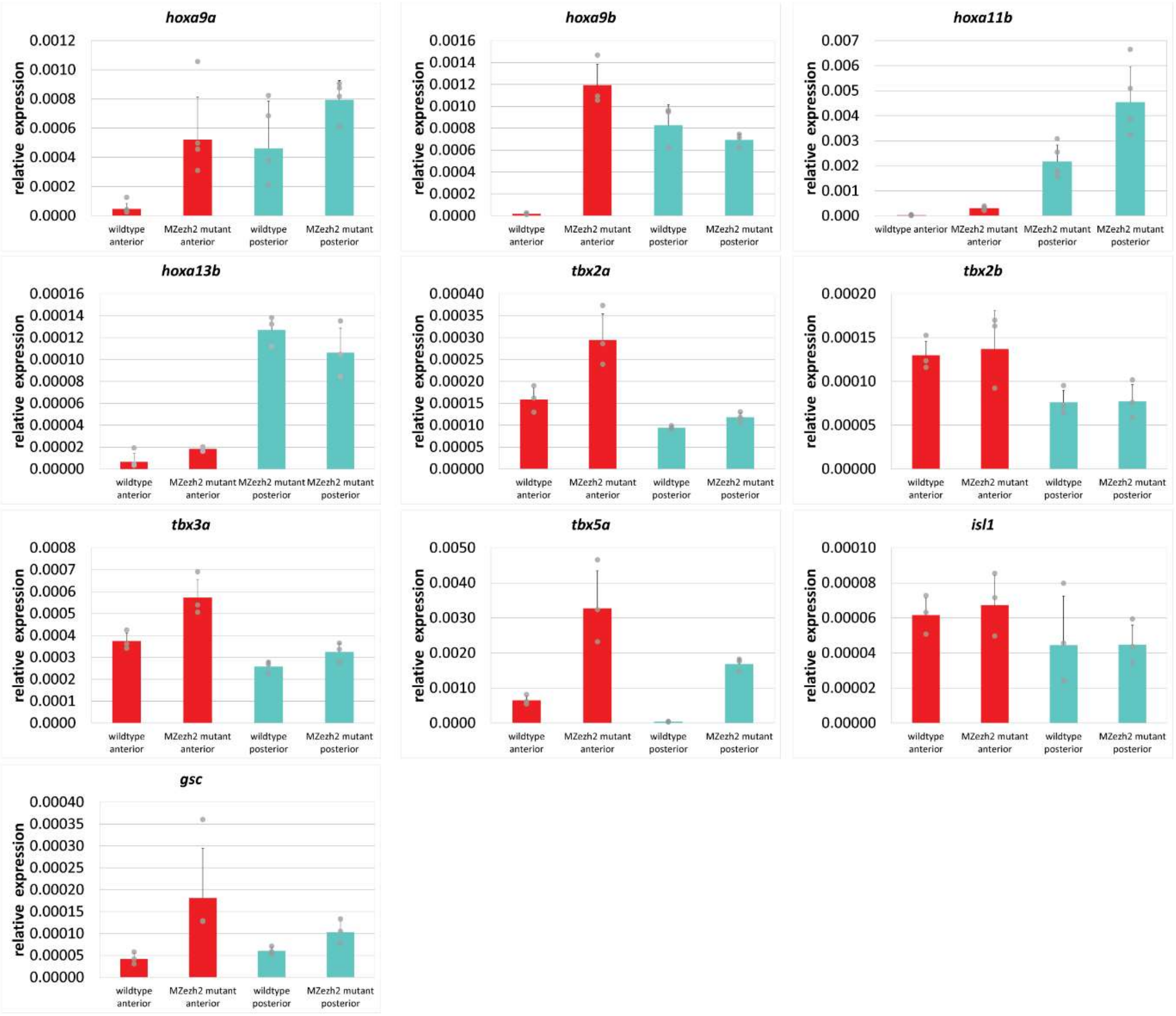

Rougeot_Supplemental_Fig.9

Fig. S9. Conclusions from RT-qPCR results of Figures 4 and 5 are similar when using alternative reference gene *eif1a*. Expression analysis of genes in Figures 4 and 5 when normalized against the reference gene *eif1a* instead of *actb1*. Bar plots represent relative expression of indicated genes in the anterior half (red) and posterior half (turquoise) of wildtype and *MZezh2* mutant (*MZezh2*$^{-/-}$) embryos at 24 hpf. Bar plots represent mean±s.e.m. and experiments were performed with at least biological triplicates. Dot plots overlaid on bar plots represent results for individual RT-qPCR samples.

Table S1. Overview of RNA-seq, ChIP-seq, and proteomics results per gene

Click here to Download Table S1

Table S2. List of primers used in this study

name	sequence	experiment	target
p3_hu5670_ComFw	CAGAATCGGTTTCCAGGTTGCCG	genotyping	*ezh2* genomic PCR
p4_hu5670_ComRv	CAGTACTCTGAGATGAACTCATTC	genotyping	*ezh2* genomic PCR
LK_ezh2_exon_Fw	TGTAAAACGACGGCCAGTCAGAATCGGTTTCCAGGTTGCCG	genotyping	*ezh2* genomic nested PCR
LK_ezh2_exon_Rv	AGGAAACAGCTATGACCATTGCAGGAGACGTTTTTACTGTCCC	genotyping	*ezh2* genomic nested PCR
Hoxa9a_RTqPCR_Fw	AAGCAGAATCTAGCCGAACTG	RT-qPCR	*hoxa9a*
Hoxa9a_RTqPCR_Rv	CACAGGGTTTTCTGGATCAGC	RT-qPCR	*hoxa9a*
Hoxa9b_RTqPCR_Fw	CAACGGATCACATGATGAGAAAAT	RT-qPCR	*hoxa9b*
Hoxa9b_RTqPCR_Rv	CCAGTTGGACGAAGGGTTA	RT-qPCR	*hoxa9b*
Hoxa11b_RTqPCR_Fw	AGCAGCAATGGACAAAAGACAC	RT-qPCR	*hoxa11b*
Hoxa11b_RTqPCR_Rv	AAGAAAAATTCTCTCTCCAGCTCT	RT-qPCR	*hoxa11b*
Hoxa13b_RTqPCR_Fw	GTGTACTGCCCGAAAGATCA	RT-qPCR	*hoxa13b*
Hoxa13b_RTqPCR_Rv	ACCTGACACGGTATCTTGGA	RT-qPCR	*hoxa13b*
tbx2a_RTqPCR_Fw	GCTAAGGAGCTTTGGGATCA	RT-qPCR	*tbx2a*
tbx2a_RTqPCR_Rv	CACCTTGAACGGAGGAAACA	RT-qPCR	*tbx2a*
tbx2b_RTqPCR_Fw	TCTCAACACATGCTTGCCTC	RT-qPCR	*tbx2b*
tbx2b_RTqPCR_Rv	AAAAGTCCACCGAAGGTTGG	RT-qPCR	*tbx2b*
tbx3a_RTqPCR_Fw	CCCGATGCCGTTTCATCTG	RT-qPCR	*tbx3a*
tbx3a_RTqPCR_Rv	CCGAAAGGAGACATAGCCAG	RT-qPCR	*tbx3a*
tbx5a_RTqPCR_Fw	GGGAGCTGATACGAGCTTTT	RT-qPCR	*tbx5a*
tbx5a_RTqPCR_Rv	CGTGAGGCCTTAAATTCCGA	RT-qPCR	*tbx5a*
isl1_RTqPCR_Fw	TTTACAAATGGCAGCAGAGC	RT-qPCR	*isl1*
isl1_RTqPCR_Rv	CGGGTTGTTTTCTCAGGTTG	RT-qPCR	*isl1*
gsc_RTqPCR_Fw	CAACAGTGTCCGTGTATTCCT	RT-qPCR	*gsc*
gsc_RTqPCR_Rv	TCATTTGATGTGGGACTGGAG	RT-qPCR	*gsc*

Development • Supplementary information

Table S3. List of antibodies used in this study

antibody	brand	ref	Concentration µg/µl	ChIP (µl/IP)	WB (dilution)
anti-Ezh2	Cell Signaling	5246S	N/A	2	1:1,000
anti-Rnf2	Cell Signaling	5694S	N/A	4	N/A
anti-H3K27me3	Millipore	07-449	N/A	2	N/A
anti-H3K4me3	Millipore	04-745	N/A	2	N/A
anti-H2A	Millipore	07-146	N/A	N/A	1:1,000
anti-Histone H3	Sigma-Aldrich	H0164	N/A	N/A	1:2,000
HRP-conjugated anti-Rabit	Dako	P0217	N/A	N/A	1:3,000

Development • Supplementary information

Table S4. Statistics for all high throughput samples generated for this study

experiment	target	genotype	time	replicate	library type	M Seqs	%aligned	M Aligned	remarks
ChIP-seq	Ezh2	*MZezh2NULL*	24 hpf	1	paired-end	1.0	35.0%	0.4	
ChIP-seq	Ezh2	*MZezh2NULL*	24 hpf	2	paired-end	33.6	51.9%	17.5	
ChIP-seq	Ezh2	*wildtype*	24 hpf	1	paired-end	23.2	71.8%	16.7	
ChIP-seq	Ezh2	*wildtype*	24 hpf	2	paired-end	34.8	56.3%	19.6	
ChIP-seq	H3K27me3	*MZezh2NULL*	24 hpf	1	paired-end	2.9	60.3%	1.8	
ChIP-seq	H3K27me3	*MZezh2NULL*	24 hpf	2	paired-end	34.7	51.0%	17.7	
ChIP-seq	H3K27me3	*wildtype*	24 hpf	1	paired-end	23.0	79.6%	18.3	
ChIP-seq	H3K27me3	*wildtype*	24 hpf	2	paired-end	31.4	68.8%	21.6	
ChIP-seq	H3K4me3	*MZezh2NULL*	24 hpf	1	paired-end	24.3	78.8%	19.2	
ChIP-seq	H3K4me3	*MZezh2NULL*	24 hpf	2	paired-end	55.6	34.6%	19.3	
ChIP-seq	H3K4me3	*MZezh2NULL*	24 hpf	3	paired-end	24.9	77.1%	19.2	
ChIP-seq	H3K4me3	*wildtype*	24 hpf	1	paired-end	40.5	76.2%	30.9	
ChIP-seq	H3K4me3	*wildtype*	24 hpf	2	paired-end	82.4	15.0%	12.4	73.9% $(TA)_n$ contamination
ChIP-seq	H3K4me3	*wildtype*	24 hpf	3	paired-end	25.8	75.4%	19.5	
ChIP-seq	INPUT	*wildtype*	24 hpf	1	paired-end	83.4	72.7%	60.6	
ChIP-seq	Rnf2	*MZezh2NULL*	24 hpf	1	paired-end	33.4	47.5%	15.9	
ChIP-seq	Rnf2	*MZezh2NULL*	24 hpf	2	paired-end	20.5	74.6%	15.3	
ChIP-seq	Rnf2	*wildtype*	24 hpf	1	paired-end	53.3	68.3%	36.4	
ChIP-seq	Rnf2	*wildtype*	24 hpf	2	paired-end	18.9	74.9%	14.2	
RNA-seq		*MZezh2NULL*	0hpf	1	single-end	32.1	83.1%	26.7	
RNA-seq		*MZezh2NULL*	0hpf	2	single-end	30.9	81.8%	25.3	
RNA-seq		*MZezh2NULL*	0hpf	3	paired-end	14.4	55.5%	8.0	
RNA-seq		*MZezh2NULL*	0hpf	4	paired-end	15.7	57.5%	9.0	
RNA-seq		*MZezh2NULL*	0hpf	5	paired-end	20.1	55.3%	10.7	
RNA-seq		*MZezh2NULL*	0hpf	6	paired-end	21.7	55.7%	12.1	
RNA-seq		*wildtype*	0hpf	1	single-end	31.2	81.4%	25.4	
RNA-seq		*wildtype*	0hpf	2	single-end	32.0	86.6%	27.7	
RNA-seq		*wildtype*	0hpf	3	paired-end	25.9	23.2%	6.0	
RNA-seq		*wildtype*	0hpf	4	paired-end	21.5	68.7%	14.8	
RNA-seq		*wildtype*	0hpf	5	paired-end	23.1	67.1%	15.5	
RNA-seq		*MZezh2NULL*	24hpf	1	single-end	32.0	84.6%	27.1	
RNA-seq		*MZezh2NULL*	24hpf	2	single-end	30.1	74.1%	22.3	
RNA-seq		*MZezh2NULL*	24hpf	3	paired-end	8.8	60.4%	5.3	
RNA-seq		*MZezh2NULL*	24hpf	4	paired-end	15.7	75.8%	11.9	
RNA-seq		*MZezh2NULL*	24hpf	5	paired-end	15.9	73.6%	11.7	
RNA-seq		*MZezh2NULL*	24hpf	6	paired-end	24.5	73.1%	17.9	
RNA-seq		*MZezh2NULL*	24hpf	7	paired-end	16.2	68.0%	11.0	
RNA-seq		*wildtype*	24hpf	1	single-end	31.0	84.5%	26.2	
RNA-seq		*wildtype*	24hpf	2	single-end	36.7	81.5%	29.9	
RNA-seq		*wildtype*	24hpf	3	paired-end	17.5	65.8%	11.5	
RNA-seq		*wildtype*	24hpf	4	paired-end	20.0	62.9%	12.6	
RNA-seq		*wildtype*	24hpf	5	paired-end	18.1	72.3%	13.1	
RNA-seq		*wildtype*	24hpf	6	paired-end	15.3	70.7%	10.8	
RNA-seq		*MZezh2NULL*	3hpf	1	single-end	33.8	79.1%	26.7	
RNA-seq		*MZezh2NULL*	3hpf	2	single-end	28.2	84.3%	23.8	
RNA-seq		*MZezh2NULL*	3hpf	3	paired-end	25.0	58.0%	14.5	
RNA-seq		*MZezh2NULL*	3hpf	4	paired-end	23.6	72.4%	17.1	
RNA-seq		*MZezh2NULL*	3hpf	5	paired-end	20.6	53.9%	11.1	
RNA-seq		*MZezh2NULL*	3hpf	6	paired-end	20.1	51.8%	10.4	
RNA-seq		*MZezh2NULL*	3hpf	7	paired-end	28.4	52.4%	14.9	
RNA-seq		*wildtype*	3hpf	1	single-end	32.9	77.5%	25.5	
RNA-seq		*wildtype*	3hpf	2	single-end	30.5	83.0%	25.3	
RNA-seq		*wildtype*	3hpf	3	paired-end	18.9	69.4%	13.1	
RNA-seq		*wildtype*	3hpf	4	paired-end	18.3	53.5%	9.8	
RNA-seq		*wildtype*	3hpf	5	paired-end	16.4	57.3%	9.4	

M Seqs = number of sequenced reads in Million   M Aligned = number of aligned reads in Million
